# Surface chemistry of metal oxide nanoparticles: NMR and TGA quantification

**DOI:** 10.1007/s00216-022-03906-x

**Published:** 2022-03-02

**Authors:** Filip Kunc, Mary Gallerneault, Oltion Kodra, Andreas Brinkmann, Gregory P. Lopinski, Linda J. Johnston

**Affiliations:** National Research Council Canada, Ottawa, ON K1A 0R6 Canada

**Keywords:** Metal oxide nanoparticles, Quantitative NMR, Thermogravimetric analysis, X-ray photoelectron spectroscopy, Quantification of surface functional groups

## Abstract

Surface functionalization is widely used to control the behavior of nanomaterials for a range of applications. However, methods to accurately quantify surface functional groups and coatings are not yet routinely applied to nanomaterial characterization. We have employed a combination of quantitative NMR (qNMR) and thermogravimetric analysis (TGA) to address this problem for commercial cerium, nickel, and iron oxide nanoparticles (NPs) that have been modified to add functional coatings with (3-aminopropyl)triethoxysilane (APTES), stearic acid, and polyvinylpyrrolidone (PVP). The qNMR method involves quantification of material that is released from the NPs and quantified in the supernatant after removal of NPs. Removal of aminopropylsilanes was accomplished by basic hydrolysis whereas PVP and stearic acid were removed by ligand exchange using sodium hexametaphosphate and pentadecafluorooctanoic acid, respectively. The method accuracy was confirmed by analysis of NPs with a known content of surface groups. Complementary TGA studies were carried out in both air and argon atmosphere with FT-IR of evolved gases in argon to confirm the identity of the functional groups. TGA measurements for some unfunctionalized samples show mass loss due to unidentified components which makes quantification of functional groups in surface-modified samples less reliable. XPS provides information on the presence of surface contaminants and the level of surface hydroxylation for selected samples. Despite the issues associated with accurate quantification using TGA, the TGA estimates agree reasonably well with the qNMR data for samples with high surface loading. This study highlights the issues in analysis of commercial nanomaterials and is an advance towards the development of generally applicable methods for quantifying surface functional groups.

**Graphical abstract:**

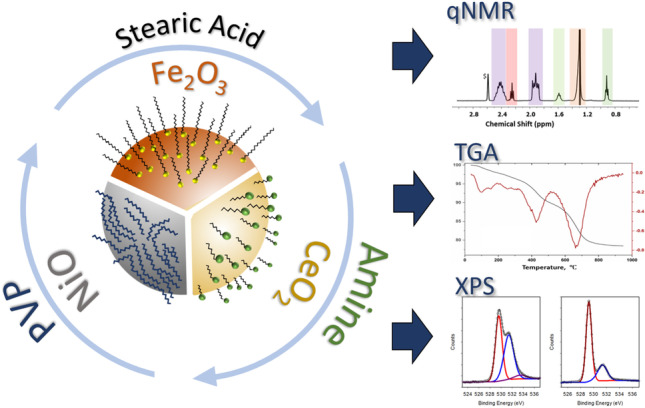

**Supplementary Information:**

The online version contains supplementary material available at 10.1007/s00216-022-03906-x.

## Introduction

Surface functional groups have a major impact on the behavior of nanomaterials since they are one of the main factors that control interactions with the surrounding environment. Despite their importance in determining the fate of nanomaterials that are used in nano-enabled products or inadvertently ingested or released to the environment, the identification and quantification of surface functional groups and coatings are considerably more challenging than measuring other important properties such as particle size distribution and composition [1–4]. Although surface chemistry is routinely used to stabilize nanomaterials against degradation and aggregation, to improve biocompatibility, and to provide functional groups for attachment of targeting moieties, it is relatively rare that surface groups are quantified, either for commercially available materials or for those produced on a laboratory scale. Nevertheless, a range of methods are available for the identification and quantification of surface functional groups [5]. These include solution- and solid-state NMR [6–13], inductively coupled plasma mass spectrometry or optical emission spectroscopy (ICP-MS, ICP-OES) [14, 15], and thermogravimetric analysis (TGA) [12, 16–18], all of which provide the total functional group content, in some cases after digestion of the material or pretreatment to remove the functional group. Other methods such as conductometric titrations [11, 19, 20] and a range of optical assays [9, 21–24] provide information on functional groups that are accessible to the assay reagents, an important factor when the nanomaterial requires further surface functionalization prior to use. Surface analysis methods such as X-ray photoelectron spectroscopy (XPS), energy-dispersive X-ray spectroscopy (EDS), and time-of-flight secondary ion mass spectrometry (ToF–SIMS) have also been employed [3, 4, 25–27].

Previous studies from our group have used a combination of optical assays, TGA, and solution quantitative NMR (qNMR) to provide estimates of the content of functional groups attached to silica nanoparticles (NPs) using amine chemistry [7, 16, 24]. The methods are complementary in that qNMR quantifies the total functional group content and also provides structural identification, whereas optical assays provide an estimate of the functional groups that are accessible to specific reagents used in the assay. TGA is primarily useful in cases where the functional group has a high molecular weight or accounts for a significant fraction of the total material (e.g., for small particles with high surface area). When combined with FT-IR of evolved gases, it can also provide information on the functional group structure and can help to separate loss of functional group from loss of adsorbed solvent or residual impurities from the synthesis of the nanomaterial. Additional work has extended the methods used for silica NPs to zinc oxides as a test case to assess feasibility for quantitative analysis of surface functional groups on metal oxides [17]. Optimized methods based on zinc oxide dissolution were developed to remove functional groups from zinc oxide NPs modified with (3-aminopropyl)triethoxysilane (APTES), caprylsilane, and stearic acid prior to qNMR quantification. A combination of TGA and solid-state NMR was used to confirm the presence of a carbonate impurity on both unfunctionalized and some surface-modified samples. XPS provided useful information on the nanomaterial surface and the metal oxidation state for both silica and zinc oxide NPs; however, quantification of organic functional groups by XPS is complicated by the presence of adventitious carbon contaminants and uncertainties in modeling the attenuation of the photoelectrons by the functional coatings for non-planar surfaces.

Here, we report the development of methods for quantification of surface functional groups on nickel, cerium, and iron oxide NPs using qNMR and TGA. This study was aimed at developing general methods that do not rely on NP dissolution to remove functional groups for qNMR quantification as well as extending the type of functional groups that can be assessed. The TGA experiments were designed to assess whether measurements under air or argon are more reliable for quantification and to use FT-IR to provide corroborating structural information with the goal of assessing the applicability of TGA for functional group quantification on metal oxide NPs. The results demonstrate the range of applicability of the qNMR and TGA methods for quantifying surface functional groups and coatings for nanomaterials with different metal oxide compositions and surface modifications.

## Materials and methods

### Materials

Unfunctionalized and surface-modified metal oxide NPs (CeO_2_, NiO, and Fe_2_O_3_) were purchased as dry powders from US Research Nanomaterials (USRN) and Sigma-Aldrich and were used as received. The sample codes, coatings, and nominal size provided by the manufacturer are provided in Table 1; the mean equivalent circular diameters measured by TEM are included for selected samples and confirm that the size provided by the supplier is reasonably accurate. Sodium hexametaphosphate (SHMP), perfluorododecanoic acid (PFDA), and 3,3,4,4,5,5,6,6,7,7,8,8,8-tridecafluorooctylphosphonic acid (PFPA) were obtained from Sigma-Aldrich and were used as received. TraceCERT maleic acid (99.94% maleic acid mass fraction) and TraceCERT potassium phthalate monobasic (99.92% mass fraction) were also purchased from Sigma-Aldrich.

**Table 1:** Unfunctionalized and surface-modified metal oxide nanoparticles (CeO_2_, NiO, and Fe_2_O_3_) used in this study

Sample code^a^	Coating	Size (supplier)	Mean equivalent circular diameter (TEM)^b^
Ce-uf	Unfunctionalized	10 nm	9.5 (0.3) nm
Ce-APTES	APTES	10 nm	-
Ce-PVP	PVP	10 nm	10.3 (0.2) nm
Ce-SA1	Stearic acid	10 nm	-
Ce-SA2	Stearic acid	10 nm	-
Ni-uf1	Unfunctionalized	18 nm	20.5 (0.8) nm
Ni-uf2	Unfunctionalized	15–35 nm	18.8 (0.5) nm
Ni-uf3	Unfunctionalized	< 50 nm	12.0 (0.7) nm
Ni-APTES	APTES	18 nm	21 (1) nm
Ni-PVP1	PVP	18 nm	17 (1)nm
Ni-PVP2	PVP	18 nm	-
Ni-SA	Stearic acid	18 nm	13.3 (0.6) nm
Fe-uf	Unfunctionalized	30 nm	34.3 (0.9) nm
Fe-APTES	APTES	30 nm	25.5 (0.5) nm
Fe-PVP	PVP	30 nm	28.0 (0.6) nm
Fe-SA1	Stearic acid	30 nm	23.7 (0.4) nm
Fe-SA2	Stearic acid	30 nm	-

^a^Samples were sourced from US Research Nanomaterials, with the following exceptions: Ni-uf3 was from Sigma-Aldrich and Ni-PVP2, Ce-SA2, and Fe-SA2 were prepared in house from Ni-uf1, Ce-uf, and Fe-uf, respectively.

^b^The standard error for the mean is provided in parentheses; the number of NPs analyzed varied from 100 to 200 for the different samples and the mean aspect ratios were between 1.2 and 1.4

The in-house modified materials were prepared by dispersing 1 g of metal oxide powder in 50 mL of ethanol by sonication in an ultrasonic bath for 10 min. This was followed by the addition of the desired quantity of functional group (e.g., 0.3 µmol of stearic acid or 0.9 µmol of PVP) and the mixture was sonicated for 10 min. Ethanol was evaporated in a rotary evaporator, and the powder was dried in high vacuum for 24 h.

### Sample preparation methods

#### APTES hydrolytic extraction

In a typical experiment, 4–12 mg of powder was weighed into an empty Eppendorf microcentrifuge tube using an analytical balance with precision ± 0.1 mg. The powder was dispersed in NaOD solution in D_2_O (0.65 mL, 0.4 M) using a short sonication step in an ultrasonic bath. Samples were placed in an orbital heated shaker and shaken at 1200 RPM at 45 °C for 24 h. The sample was then cooled to room temperature and centrifuged at 18 k rcf for 5 min and the supernatant (typically 0.6 mL) was separated from the pellet using a calibrated pipettor. In some cases, a new aliquot of NaOD solution was added to the recovered pellet and the process was repeated, indicated as a “2^nd^” wash. Prior to the qNMR experiment, a solution of the internal standard, potassium hydrogen phthalate in D_2_O, was added to the sample. The data for amine content was corrected for the incomplete removal of the supernatant.

#### PVP desorption

In a typical experiment, 5–10 mg of powder was weighed into an empty Eppendorf microcentrifuge tube. The powder was dispersed in 0.65 mL of D_2_O or 0.65 mL sodium hexametaphosphate solution (0.5% w/w in D2O) using a short sonication step. The sample was placed in an orbital heated shaker and shaken at 1200 RPM at 45 °C for 24 h. The sample was then cooled to room temperature and centrifuged at 18 k rcf for 5 min and the supernatant was separated from the pellet. In some cases, a new aliquot of D_2_O was added to the recovered pellet and the process was repeated, indicated as a “2^nd^” or a “3^rd^” wash. Prior to the qNMR experiment, a solution of the internal standard, potassium hydrogen phthalate in D_2_O, was added to the sample.

#### Stearic acid solvent desorption and ligand exchange

In a typical experiment, 4–12 mg of powder was weighed into an empty Eppendorf microcentrifuge tube. The powder was dispersed in 0.65 mL of DMSO-d_6_ for solvent extraction or a CD_3_OD solution of perfluoralkyl reagent (PFDA or PFPA) for the ligand exchange experiments. The powders were dispersed by a short sonication step. Samples were placed in an orbital heated shaker and shaken at 1200 RPM at 45 °C for 24 h. The sample was cooled to room temperature and centrifuged at 18 k rcf for 5 min and the supernatant was separated from the pellet. In some cases, a new aliquot of either DMSO-d_6_ or CD_3_OD was added to the recovered pellet and the process was repeated, indicated as a “2^nd^” or a “3^rd^” wash. Prior to the qNMR experiment, the sample was combined with a solution of maleic acid internal standard in DMSO-d_6_.

### qNMR

qNMR experiments were carried out at 20 °C (± 1 °C) with an Avance III 400 MHz spectrometer equipped with a 5-mm Bruker BBFO probe with one proton channel connected to the outer radio frequency (rf) coil, one broadband channel connected to the inner rf coil, and one deuterium lock channel. Calibrations and relaxation times were obtained as previously described [7, 17]. The relaxation delays for the measurement were set at 7 × the highest relaxation time of the analyzed protons. These corresponded to internal standards (6.1 s for maleic acid, and max 2.5 s for potassium hydrogen phthalate in D_2_O), as provided by the manufacturer. The relaxation times of the analyte alkyl protons were experimentally verified to be less (0.96–1.22 s) than those of the internal standard. ^1^H-NMR spectra were recorded using a 90° pulse program with the following parameters: two dummy transients, 16–32 transients, and 20.0 ppm spectral width with 6.1 ppm transmitter offset. Spectra were processed by Fourier transformation and phase- and baseline-corrected manually using a fourth-order polynomial fit; the correction is done over an area around the peak that is slightly larger than the integration region. Analyte signals were identified and integrated and normalized for the number of protons. Signals for which the integrals deviated by > 20% from the average of other integrals were excluded; specifically, the CH_3_ signal for one sample of both cerium and iron oxide NPs was broadened with a small shoulder and was not used for quantification. The average of the remaining integrals was used to calculate the final content (µmol) of functional group per gram of material; the final values are the average of individual replicates, each on an independently prepared sample, with standard deviation.

### TGA

TGA experiments were run on either a NETZSCH Iris TG209 F1 or a NETZSCH Jupiter STA449 F1 instrument coupled to a Bruker Tensor 27 FT-IR spectrometer. Temperature and mass calibrations were carried out using the manufacturer’s recommended procedures. Twenty to forty milligrams of dry powdered sample was loaded in an empty aluminum oxide crucible that was pre-treated by annealing for ~ 30 s. The sample mass was adjusted to ensure a total mass loss of > 1 mg. The sample was inserted in the instrument under 50 mL min^−1^ argon (or air) atmosphere (argon protective 25 mL min^−1^) and stabilized for 1 h; the FT-IR transfer line was also purged with the same flow of argon/air. The thermal cycle 25–950 °C (10 °C min^−1^) was then initiated maintaining the same gas flow. For FT-IR results, the residence time in the transfer line is ∼2.5 s. All TGA experiments were run against the correction for an empty aluminum oxide crucible in an atmosphere of argon/air. Thermograms were processed by excluding the mass loss below ∼200 °C due to the presence of water. All TGA figures show both mass loss and the derivative curve (DTG). The position of maximum mass loss from the DTG curve is reported to identify the various components and mass loss values are reported as mass %. The mass loss region assigned to the functional group or surface coating is noted in the text and is selected so as to exclude contributions at low temperatures (predominantly water, < 200 °C) and unidentified components at higher temperatures.

### XPS

XPS measurements were done on an Axis Ultra DLD spectrometer (Kratos Analytical) with monochromatized Al Kα X-rays. Survey spectra over the entire energy range were first obtained in order to estimate the relative atomic composition of the sample and detect any impurities that may be present. High-resolution spectra were subsequently acquired in regions corresponding to the strongest core level transitions for the major elements present on these samples. Data analysis was carried out with the CasaXPS software (Casa Software Limited). Atomic composition was determined from the integrated intensities of the major core level regions, subtracting a Shirley background and employing Kratos relative sensitivity factors. Decomposition of the C1s and O1s spectra into various components was carried out using mixed Gaussian–Lorentzian (GL30) lineshapes.

## Results and discussion

### qNMR of APTES-modified NPs

**Fig. 1:**
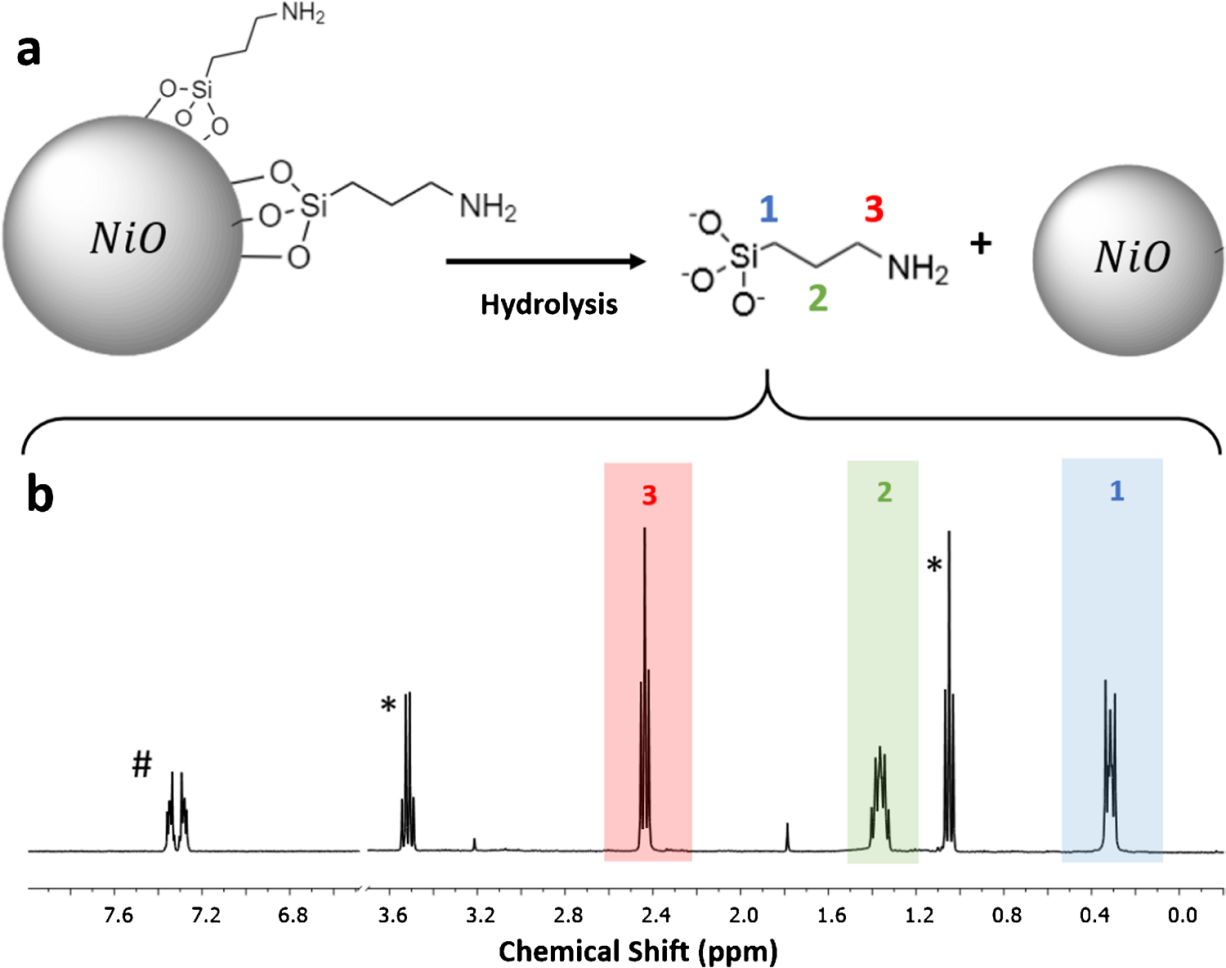
(**a**) Reaction for removal of aminopropyl silane from Ni-APTES NPs by basic hydrolysis in 0.4 M NaOD for 24 h at 45 °C. The procedure solubilizes aminopropylsilane into the deuterated solvent while the metal oxide NPs remain intact and are removed by centrifugation. (**b**) ^1^H NMR spectrum of the supernatant with quantification by comparison to the internal standard (#); the region with the H–O signal between 3.7 and 6.5 ppm was removed for clarity and the full spectrum is shown in Fig. S1 of the Electronic Supplementary Material (ESM)

Our previous study of APTES-modified zinc oxide NPs concluded that hydrolysis of the functional group in basic solution provided reproducible results for release of 3-aminopropylsiloxane from the surface for quantitation by qNMR [17]. This is a more general method than complete dissolution of the NP, as used previously for silica NPs [7], since the ease of dissolution of various metal oxides varies significantly. A similar procedure (hydrolysis in aqueous 0.4 M NaOD for 24 h at 45 °C, followed by pelleting of the NPs and analysis of the supernatant) was followed for the three metal oxides studied here. A reaction scheme and a representative ^1^H NMR spectrum for APTES-functionalized nickel oxide NPs are shown in Fig. 1a and b, respectively. The spectrum is typical of APTES-modified NPs for all three metal oxides and shows three signals (denoted as 1, 2, and 3) corresponding to the propylamine chain, and signals of residual ethanol (denoted as *) and the internal standard (#). The presence of ethanol may originate from the incomplete hydrolysis of all three APTES ethoxy groups during the surface functionalization [28] or from incomplete solvent removal after the APTES reaction which is typically carried out in ethanol.

The results for quantification of functional group content on the three metal oxide NPs are summarized in Table 2. Both Ce-APTES and Ni-APTES had high loadings (1055 and 633 µmol/g, respectively) of functional group. An additional hydrolysis step for Ni-APTES resulted in removal of only a trace (11 µmol/g, < 0.2%) of additional functional group and the data for Fe-APTES were obtained at both 45 °C and 80 °C with an ~ 10% higher yield at the higher temperature. Overall, we conclude that hydrolysis in basic solution at 45 °C for 24 h is a generally applicable route for removal of siloxane groups from APTES-modified metal oxide NPs. Note that removal of silanes with larger alkyl groups may require a modified procedure using basic methanol to solubilize more hydrophobic groups and/or an additional hydrolysis step as used previously for caprylsilane-modified ZnO NPs [17]. Table 2 also provides the APTES content in coverage/surface area (molecules/nm^2^), which is useful for later comparisons. This was calculated from the qNMR data and the nominal NP diameter and metal oxide density provided by the manufacturer. Based on this estimate, the surface coverage varies by a factor of 3 for the APTES-modified samples.

**Table 2:** Functional group content measured by qNMR after hydrolysis, solvent wash, or ligand exchange at 45 °C (unless otherwise noted) to remove surface ligands from CeO_2_, NiO, and Fe_2_O_3_ NPs

Sample code	Functional group content, µmol/g^a^			Molecules/nm^2 b^
	Basic hydrolysis			
Ce-APTES	1055 ± 12 (*n* = 2)			7.6
Ni-APTES	633 ± 33 (*n* = 2)			7.6
Fe-APTES	152 ± 4^c^ (*n* = 2)			2.4
	D_2_O wash	Ligand exchange, 0.5% SHMP/D_2_O		
Ce-PVP	688 ± 29 (*n* = 2) 70 (2^nd^ wash)^d^	916 ± 25 (*n* = 2)		
Ni-PVP1	612 (*n* = 1) 18 (2nd wash)^d^	880 ± 60 (*n* = 2)		
Ni-PVP2^e^	734 (*n* = 1)	863 (*n* = 1)		
Fe-PVP	398 ± 11^f^ (*n* = 2)	358 ± 55 (*n* = 2)		
	Ligand exchange, 3.5 µmol PFDA	Ligand exchange, 35 µmol PFDA	Ligand exchange, 3.5 µmol PFPA	
Ce-SA1	191 ± 19 (*n* = 3)	229 ± 116 (*n* = 5)	414 ± 86 (*n* = 3)	1.6
Ce-SA2^g^	193. ± 74 (*n* = 3)	256 ± 12 (*n* = 3)	251 ± 10 (*n* = 3)	1.8
Ni-SA	544 ± 36 (*n* = 2)	457 ± 20 (*n* = 3)	561 ± 34 (*n* = 3)	5.5
Fe-SA1	114 ± 5.2 (*n* = 3)	162 ± 14 (*n* = 3)	156 ± 9 (*n* = 3)	2.6
Fe-SA2^g^	208 (*n* = 1)	260 (*n* = 1)	267 (*n* = 1)	4.1

^a^The number of replicates, each of which is an independently prepared sample, is shown in parentheses. The PVP data is per monomer unit.

^b^The functional group content in molecules/nm^2^ is based on qNMR data for basic hydrolysis (APTES) and ligand exchange with 35 µmol PFDA (stearic acid).

^c^Extraction at 80 °C: 168 ± 12 µmol/g.

^d^A third wash had PVP that was either undetectable or below the limit of quantification.

^e^Prepared using 900 µmol/g PVP.

^f^Extraction at 80 °C.

^g^Prepared using 300 µmol/g stearic acid.

### qNMR of PVP-coated NPs

**Fig. 2:**
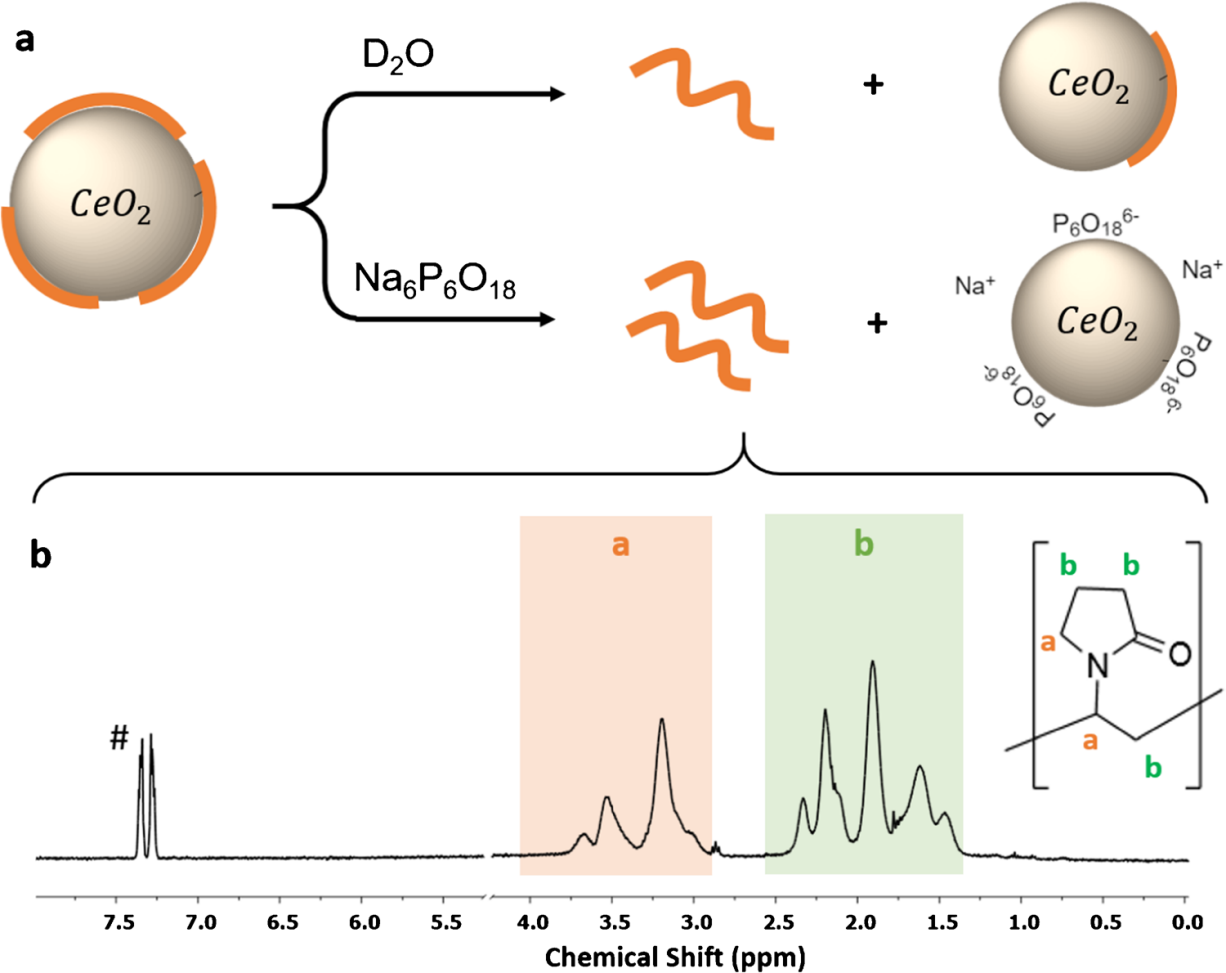
**a** Reaction for PVP removal from Ce-PVP by a D_2_O wash and ligand exchange with 0.5% SHMP. The ^1^H NMR spectrum obtained using the SHMP ligand exchange method is shown in (**b**)

Initial tests to remove PVP from metal oxides for qNMR analysis used a solvent wash method (Fig. 2a) which equilibrated the material in D_2_O at 45 °C, centrifuged the sample to pellet the NPs, and analyzed the supernatant (Table 2). A second D_2_O wash step removed as much as 10% of the PVP recovered in the first solvent extraction step for both Ce-PVP and Ni-PVP1; the PVP signal was absent (below limit of detection) after a third wash step. This demonstrated that a second wash was required for quantitative removal of PVP. Therefore, a ligand exchange method using sodium hexametaphosphate (SHMP) was also tested (Fig. 2a). SHMP has been used as a ligand to stabilize nanomaterials and to improve their dispersibility [29, 30], suggesting that an excess of SHMP could be used to displace PVP from the surface of metal oxide NPs without adding any NMR-active material to interfere with quantitation. The method was tested by preparing a PVP-modified NiO sample (Ni-PVP2) with a known PVP content and using ligand exchange with 0.5% SHMP to remove the PVP. This demonstrated recovery of 96% of the initial PVP after ligand exchange and collection of the supernatant for NMR analysis (Table 2). By contrast, a single D_2_O wash step removed only 85% of the initial PVP (Table 2).

Representative NMR spectra obtained by using SHMP ligand exchange to remove PVP from Ce-PVP and Ni-PVP1 are shown in Fig. 2b and Fig. S2a of the ESM, respectively. The PVP signal regions labelled a and b represent the average chemical environments of the vinylpyrrolidone group in the polymer. Figure S2b also shows the spectrum obtained using a D_2_O wash; in this case, the signal due to internal standard is broadened and there is a slight shift in the PVP signals. This effect was observed for both Ni-PVP samples, but not for Ni-APTES or CeO_2_ or Fe_2_O_3_ samples modified with either APTES or PVP. These changes can be attributed to the presence of residual paramagnetic impurities (Ni ions or NPs) and possible coordination of Ni to the internal standard. The PVP content for all samples is summarized in Table 2. The SHMP procedure gave good repeatability for removal of PVP from both Ce-PVP and Ni-PVP1, with higher yields of PVP than were obtained using either one or two D_2_O wash steps. Ligand exchange with SHMP gave higher variability (relative standard deviation of 22%) for Fe-PVP (Table 2).

### qNMR of stearic acid-coated NPs

Several solvent extraction and dissolution methods were tested previously to remove stearic acid from ZnO NPs [17]. Although acidic dissolution of ZnO NPs using trifluoroacetic acid in methanol-d_4_ gave the best results, the repeatability was lower than for removal of siloxane functional groups. Since the rate of dissolution of different metal oxides varies significantly [31], here, we tested both solvent wash and ligand exchange methods as alternatives to dissolution of the metal oxide core. Solvent extraction using DMSO gave low recovery of stearic acid, even after several extraction steps for Ce-SA1 although higher values were obtained for Ni-SA and Fe-SA1. However, tests on in-house modified CeO_2_ and Fe_2_O_3_ NPs with a known stearic acid content demonstrated that ≤ 15% of the initial stearic acid was recovered in a single wash step (ESM, Table S1). Based on the low recovery for these samples and the variable recovery for the commercial samples, this method was not pursued further. Ligand exchange with SHMP was not useful in this case due to the limited solubility of SHMP in DMSO and methanol.

**Fig. 3:**
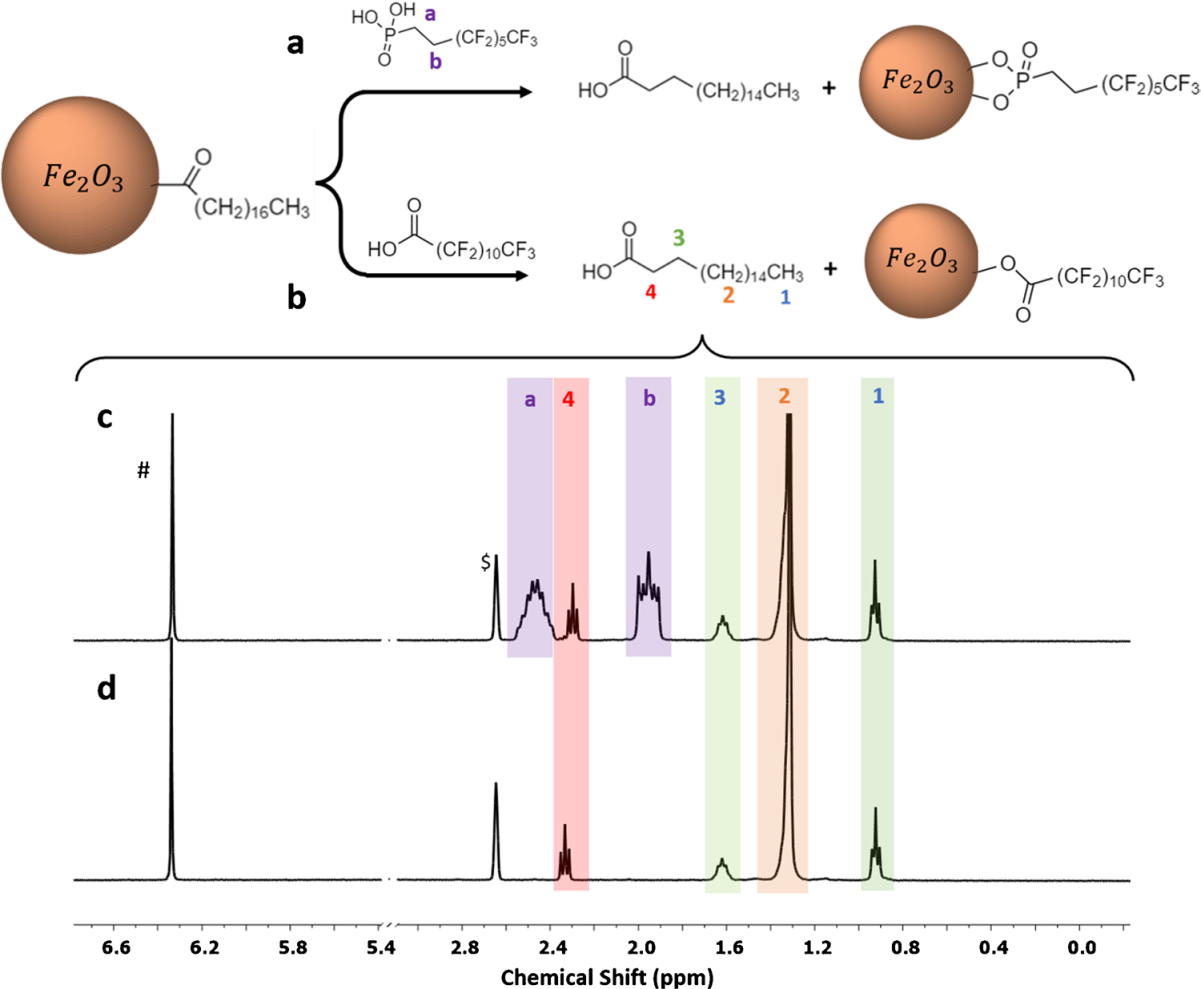
Stearic acid removal from the NP surface by exchange with PFPA (**a**) and PFDA (**b**). (**c**) and (**d**) show ^1^H NMR spectra obtained by ligand exchange for Fe-SA1 NPs using 0.0054 M PFPA and 0.054 M PFDA, respectively. Note that signals “a” and “b” in (**c**) correspond to the non-fluorinated methylenes of PFPA and $ corresponds to DMSO from the addition of internal standard maleic acid denoted as #

**Fig. 4:**
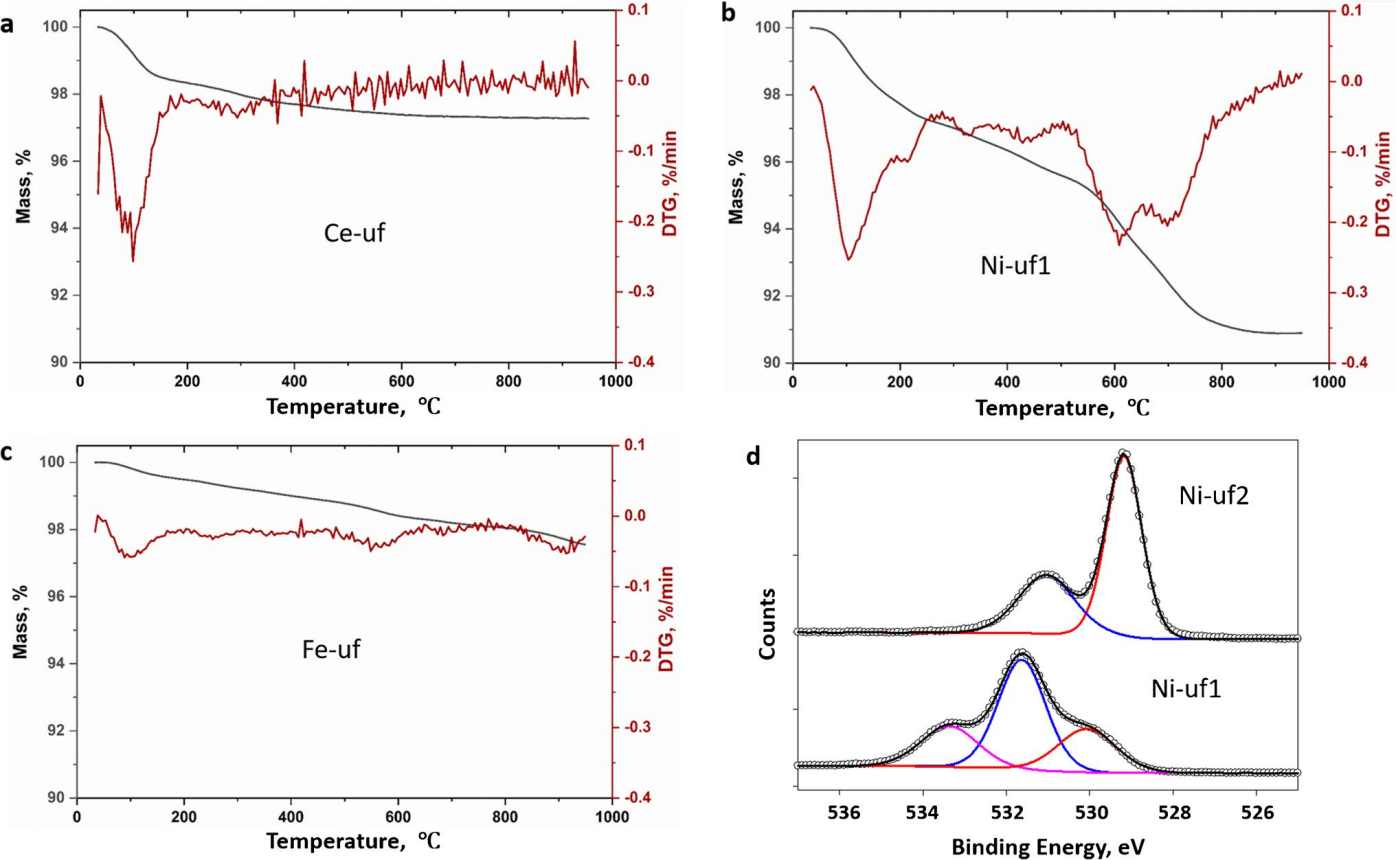
Representative TGA results for unfunctionalized metal oxides measured in an argon atmosphere: (**a**) CeO_2_, (**b**) NiO (Ni-uf1), note the large mass loss above 550 °C, and (**c**) Fe_2_O_3_. Panel (**d**) shows the XPS O1s region for two NiO NP samples (Ni-uf1, Ni-uf2) that have different levels of surface hydroxyl content

**Fig. 5:**
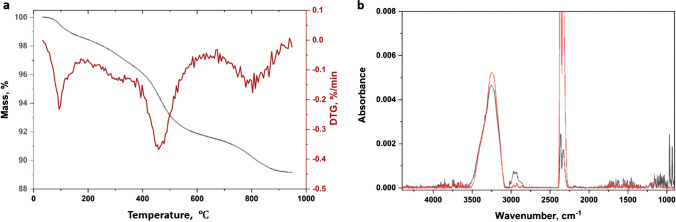
Representative TGA results (**a**) for Ce-APTES measured in an argon atmosphere and FT-IR spectra of evolved gases measured at 455 °C (**b**, black) and 790 °C (**b**, red). The signal between 3000 and 3500 cm^−1^ is due to ice condensation in the detector

**Fig. 6:**
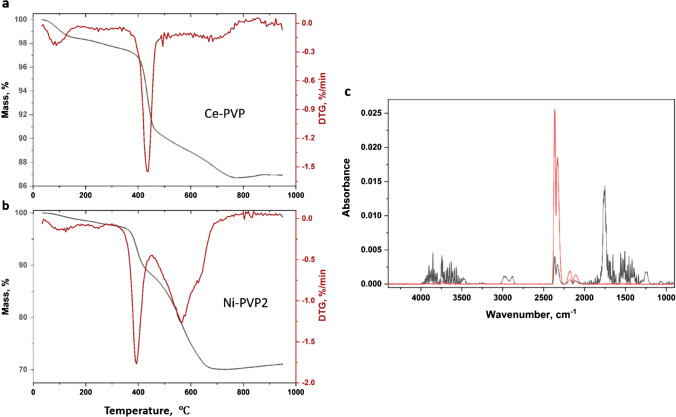
Representative TGA results (**a**) for Ce-PVP (**a**) and Ni-PVP2 (**b**) measured in an argon atmosphere and FT-IR spectra of evolved gases for Ni-PVP2 measured at 390 °C (c, black) and 580 °C (c, red)

**Fig. 7:**
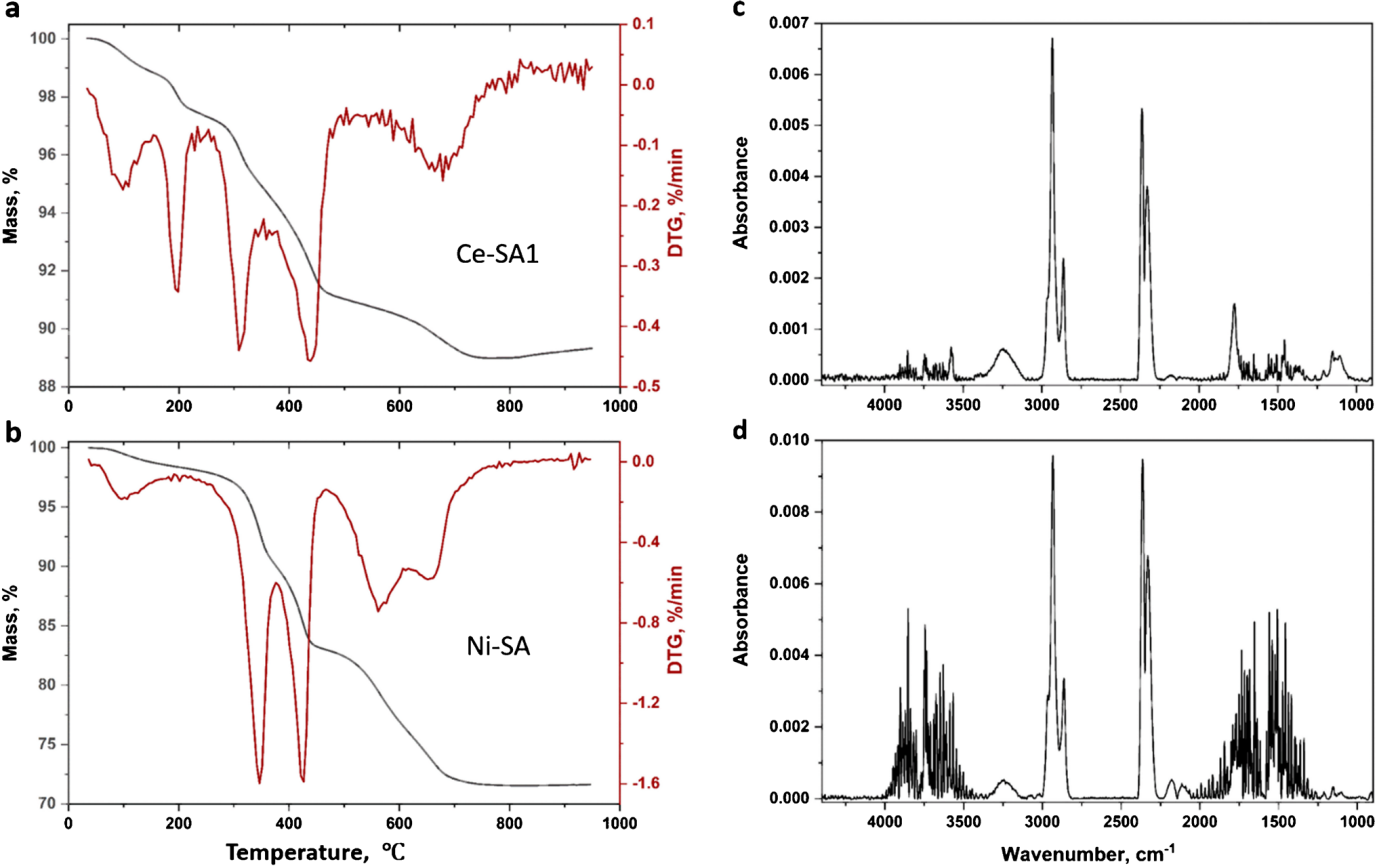
Representative TGA results for Ce-SA1 (**a**) and Ni-SA (**b**) measured in an argon atmosphere and FT-IR spectra of evolved gases for Ni-SA measured at 330 °C (**c**) and 420 °C (**d**)

**Fig. 8:**
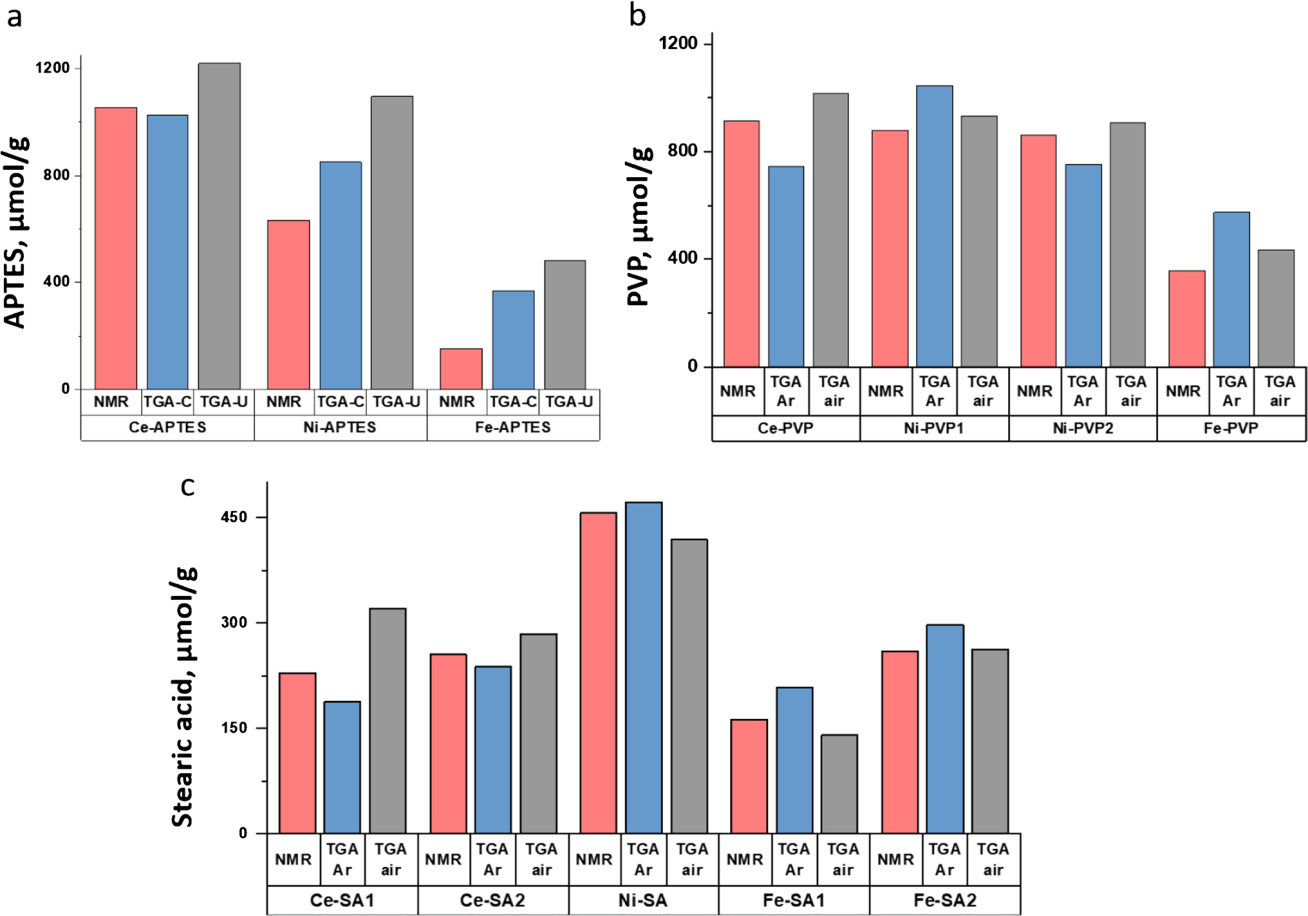
Comparison of qNMR and TGA data for surface functional group content in CeO_2_, NiO, and Fe_2_O_3_ NPs: (**a**) APTES, (**b**) PVP, and (**c**) stearic acid. The TGA data in (**a**) shows uncorrected (TGA-U) data as well as estimates after correction (TGA-C) for mass loss in an unfunctionalized sample of equivalent size from the same supplier. The TGA data for (**b**) and (**c**) show functional group content from thermograms recorded under argon and air after correction for an unfunctionalized sample of equivalent size

Motivated by a recent study of the surface binding of phosphonic acids and carboxylic acids to form self-assembled monolayers on titanium dioxide NPs [32], we next investigated ligand exchange using both perfluorododecanoic acid (PFDA, Fig. 3a) and 3,3,4,4,5,5,6,6,7,7,8,8,8-tridecafluorooctylphosphonic acid (PFPA, Fig. 3b) in CD_3_OD. Three conditions were tested: PFDA at concentrations of 0.054 and 0.0054 M and PFPA at 0.0054 M. Note that 0.0054 M PFDA or PFPA corresponds to a 1:1 ratio with stearic acid for a typical 7-mg sample with a stearic acid loading of 500 µmol/g. Based on the previous study [32], one would predict incomplete exchange for the lower concentration of PFDA, but close to complete exchange for the higher concentration and for the phosphonic acid which has a much higher adsorption constant than carboxylic acids [32]. Representative spectra are shown in Fig. 3c and d for stearic acid-modified iron oxide (Fe-SA1). The spectra in the presence of PFPA have signals due to the two unfluorinated methyl groups (a, b) on residual PFPA in the supernatant, as well as stearic acid.

The quantification of stearic acid for the various experiments for the three metal oxides is summarized in Table 2. There are several things to note. First, the stearic acid yield is lower for the lower concentration of PFDA than for either the higher PFDA concentration or PFPA; the latter two conditions give similar yields for all samples with the exception of Ni-SA, for which a lower value is obtained for the higher PFDA concentration. Second, the recovered stearic acid is ≥ 85% of the initial amount used for the two in-house modified samples that were analyzed. The third observation is that there is good repeatability for most measurements, with standard deviations on the order 4–9% for samples using high [PFDA] or PFPA. There is one exception for Ce-SA1, for which high variability in measurements for all three sets of ligand exchange conditions was observed. This does not appear to be a general problem for cerium oxides since the in-house modified sample (Ce-SA2) gave 85% recovery with a low standard deviation using the higher concentration of PFDA. The source of the poor repeatability remains unclear, despite attempts to ensure adequately dispersed samples by adding a probe sonication step and to test several different temperatures for the ligand exchange. Finally, the surface coverage (molecules/nm^2^) varies by a factor of ~ 3.5 for these samples, similar to the variation observed for the APTES-modified NPs.

An additional issue was observed when PFPA was used for the ligand exchange for Ni-SA. In this case, the methylene signals of PFPA were unusually broadened in the NMR spectrum (see Fig. S3). We examined this phenomenon by a series of control experiments to rule out possible interactions between reagents and NiO (Fig. S4). However, it appears that the broadening can be attributed to the prolonged exposure of NiO to PFPA (Fig. S5). We hypothesize that under these conditions, the phosphonic acid may partially dissolve the NiO NPs, and the distortion of the spectra can be attributed to nickel ions present in the supernatant (see discussion in the ESM). Dissolution of NiO NPs has been observed in water at neutral pH, cell culture media, and artificial lysosomal fluid (pH 4.5) [33–35]. Based on these observations, we conclude that ligand exchange with an excess of PFDA (PFDA/stearic acid of ~ 10:1) is the preferred method to remove stearic acid since it avoids additional methylene signals in the NMR spectrum for PFPA and shows no evidence of complications due to partial dissolution of the NP core. The values obtained using this method are summarized in Table 2.

### TGA of unfunctionalized NPs

Thermograms were measured for unfunctionalized CeO_2_, NiO, and Fe_2_O_3_ NPs of the same size and from the same supplier as the surface-modified NPs studied by qNMR and TGA. Previous TGA studies have shown the importance of examining unfunctionalized samples in order to account for mass losses that are not attributable to the surface functional group or coating [16, 17]. Representative TGA results for samples run in an argon atmosphere are shown in Fig. 4. All samples have a mass loss around 100 °C consistent with loss of water. CeO_2_ shows low mass loss (~ 1%) between 200 and 900 °C. Unfunctionalized Fe_2_O_3_ NPs gave similar results with a slightly higher mass loss of ~ 1.8% under argon or air. By contrast, Ni-uf1 showed distinct mass loss peaks at higher temperatures (> 250 °C) under argon with the largest mass loss between 550 and 850 °C. The total mass loss after excluding the water component was between 6 and 7% in both air and argon atmosphere for this sample.

Thermograms for two additional NiO samples were measured to determine whether the large mass loss for Ni-uf1 was typical (Fig. S6). A second sample with a slightly different size (Ni-uf2, 15–35 nm) from the same supplier gave a low mass loss (1.1% from 200 to 900 °C) with no distinct peaks in the DTG curve; the large mass loss starting at ~ 550 °C for Ni-uf1 was not observed. A third sample (Ni-uf3, Sigma, 30 nm) also had low mass loss between 330 and 950 °C (1.5%), plus a larger water peak at ~ 100 °C. These two observations indicate that the large mass loss for Ni-uf1 is not typical of all NiO NPs. FT-IR spectra of evolved gases from Ni-uf1 (Fig. S7) and Fe-uf showed predominantly loss of water and CO_2_, which is not particularly diagnostic. Ni-uf1 also had unidentified signals between 1200 and 1800 cm^−1^ above 800 °C (data not shown).

XPS measurements were carried out for the three unfunctionalized NiO NPs in an attempt to identify the contaminant observed for Ni-uf1. First, Br was detected in survey scans of Ni-uf1 (2.2% atomic composition), but not for the other two samples. Second, examination of the O1s region showed that Ni-uf2 had a strong signal due to lattice oxygen at 529.2 eV and a weaker hydroxyl signal (531 eV) (Fig. 4d). In contrast, Ni-uf1 had a large hydroxyl signal (531.6 eV) and weaker signals due to lattice oxygen (530.1 eV) and water (533.4 eV). The increased hydroxyl signal agrees with an earlier XPS study of nano-sized and bulk NiO before and after prolonged exposure to ambient conditions which resulted in extensive hydroxylation and water absorption [36]. We hypothesize that the large mass loss for Ni-uf1 may result from impurities from the synthesis that are not removed during purification. The detection of Br (by XPS) in this sample suggests the use of a different synthetic route that may also result in a higher level of surface hydroxylation. Note that FT-IR spectra show the loss of water, primarily below 200 °C for Ni-uf1 (Fig. S7a), some of which may come from surface hydroxyls; however, there is no indication in the FT-IR spectra (Fig. S7b) that water accounts for the large mass loss above 500 °C. Interestingly, Ni-uf1 is unusual in that it has a large peak due to C-O species in the C1s region. In contrast, the other unmodifed samples have varying levels of carbon contamination, but the C–C signal predominates. The presence of C-O contaminants supports the conclusion from FT-IR that dehydration of surface hydroxyls is not responsible for the high temperature mass loss. Overall, the combined TGA and XPS results are consistent with a significant (unidentified) contaminant that probably comes from the synthesis. The variable results for NiO are similar to literature TGA studies for NiO NPs prepared from various precursors which report loss of water by dehydration of surface hydroxyls and loss of impurities over various temperature ranges [37–39].

### TGA of APTES-modified NPs

The representative thermogram for Ce-APTES (Fig. 5a) has the largest mass loss at 455 ℃ with an additional smaller mass loss at high temperature (~ 800 °C). FT-IR spectra of evolved gases at 455 °C show loss of CO_2_ and weak bands at 2800–3000 cm^−1^ and ~ 900 cm^−1^, respectively, that are consistent with library spectra for alkyl groups and ammonia (Fig. 5b, black). The mass loss occurs in a similar temperature range as observed previously for APTES-functionalized ZnO (maximum at 400 °C [17]); FT-IR of evolved gas for this sample did not show the presence of alkyl groups, presumably due to the low aminopropylsilane content and low sensitivity of gas phase FT-IR. The evolved gas FT-IR spectrum at higher temperatures is dominated by CO_2_ and background signal due to ice condensation in the detector (Fig. 5b, red). Thermograms for Ni-APTES (Fig. S8a) show a similar mass loss at 420 °C that can be assigned to loss of functional group and a large mass loss at 670 °C, similar to that observed for the unfunctionalized sample, Ni-uf1. The FT-IR in this case shows mainly loss of CO_2_. For Fe-APTES (Fig. S8b), the main peak is shifted to 395 ℃, similar to ZnO, and there is low mass loss at higher temperature.

The mass loss in the intermediate temperature range (approximately between 200 and 600 °C which includes a shoulder at lower temperature, but not the high mass loss region) was used to quantify the functional group content. Table 3 summarizes the results, with estimates of functional group loading based on loss of only propylamine before and after correction for mass loss in the unfunctionalized sample of the same size. Note that the sample correction is not necessarily the optimal approach for commercial samples with an unknown sample history. This approach also ignores possible contributions of residual ethoxy groups that may be present due to incomplete APTES hydrolysis during the surface modification procedure. If some of the ethanol detected by NMR does arise from unreacted ethoxy groups, the amount of functional group will be lower than the estimates provided in Table 3.

**Table 3:** Quantification of surface ligands on CeO_2_, NiO, and Fe_2_O_3_ NPs by TGA

Sample code	Functional group content, µmol/g^a^			
	TGA, argon corrected	TGA, argon uncorrected	TGA, air corrected	TGA, air uncorrected
Ce-APTES	1026 ± 10 (*n* = 2)	1221 ± 11 (*n* = 2)		
Ni-APTES	851 (*n* = 1)	1096 (*n* = 1)		
Fe-APTES	370 (*n* = 1)	481 (*n* = 1)		
Ce-PVP	744 ± 22 (*n* = 2)	788 ± 23 (*n* = 2)	1017 (*n* = 1)	1131 (*n* = 1)
Ni-PVP1	1046 ± 45 (*n* = 2)	1137 ± 46 (*n* = 2)	934 (*n* = 1)	1381 (*n* = 1)
Ni-PVP2^b^	753 (*n* = 1)	848 (*n* = 1)	909 ± 1 (*n* = 2)	1308 ± 1 (*n* = 2)
Fe-PVP	574 ± 10 (*n* = 2)	633 ± 10 (*n* = 2)	436 (*n* = 1)	520 (*n* = 1)
Ce-SA1	188 ± 12 (*n* = 3)	213 ± 12 (*n* = 3)	321 (*n* = 1)	347 (*n* = 1)
Ce-SA2 ^*c*^	238 (*n* = 1)	263 (*n* = 1)	284 (*n* = 1)	309 (*n* = 1)
Ni-SA	472 ± 3 (*n* = 3)	528 ± 3 (*n* = 3)	419 (*n* = 1)	585 (*n* = 1)
Fe-SA1	209 ± 2 (*n* = 3)	230 ± 2 (*n* = 2)	141 ± 7 (*n* = 2)	162 ± 7 (*n* = 2)
Fe-SA2^c^	298 (*n* = 1)	319 (*n* = 1)	263 (*n* = 1)	290 (*n* = 1)

^a^The number of replicates, each of which is an independently prepared sample, is shown in parentheses. The PVP data is per monomer unit.

^b^Prepared using 900 µmol/g PVP.

^c^Prepared using 300 µmol/g stearic acid.

The aminopropylsilane content on the surface was compared to the nitrogen content determined by XPS. The nitrogen content estimated from XPS was 5.7 ± 0.5% for Ce-APTES, 6.0 ± 0.2% for Ni-APTES, and 2.3 ± 0.1% for Fe-APTES. This trend in nitrogen content is in remarkably good agreement with the estimated surface coverages from the qNMR data of 7.6 molecules/nm^2^ for the APTES-modified CeO_2_ and NiO NPs and 2.4 molecules/nm^2^ for Fe_2_O_3_ NPs (Table 2). Although multiple sizes or batches of the modified metal oxide particles have not been studied in this work, previous studies of APTES-functionalized silica have shown that the batch-to-batch variation in siloxane content for the same size NP from a single supplier is as high as a factor of 5 and estimated surface coverages for samples with different sizes from different suppliers vary from a low of 10–20% to over 100% [7]. Similar conclusions were drawn from a more limited number of samples for ZnO NPs [17] and it is likely that this is also the case for metal oxide NPs studied here.

### TGA of PVP-coated NPs

Thermograms under both argon and air were measured for four samples modified with PVP and FT-IR spectra of evolved gases were measured for each sample under argon. Each sample showed a large mass loss around 400 °C under argon with the maximum at slightly different positions for the three metal oxides. Representative thermograms are shown in Fig. 6a and b for Ce-PVP and Ni-PVP2, respectively. The peaks at ~ 400 °C account for most of the mass loss for both Ce-PVP and Fe-PVP (data not shown). By contrast, the two NiO samples (Ni-PVP1 and Ni-PVP2) had additional mass loss at higher temperatures, similar to thermograms for the corresponding unfunctionalized sample (Ni-uf1). The FT-IR spectrum of evolved gases at 390 °C for Ni-PVP2 is shown in Fig. 6c (black) and has a signal in the carbonyl region consistent with assignment to the PVP carbonyl, as well as weak signals at 2800–3000 cm^−1^ that can be assigned to alkyl groups and traces of water. With the exception of Fe-PVP, the other samples show similar spectra for the main 400 °C peak with a clear peak in the carbonyl region. Spectra at higher temperatures for both NiO samples and Ce-PVP showed a strong peak due to CO_2_ and signals between 2000 and 2300 cm^−1^ that are consistent with the presence of C≡C bonds (Fig. 6c, red).

Based on the above results, the mass loss at ~ 400 °C can confidently be assigned to PVP degradation and was used to quantify the polymer content; signals below 200 and at higher temperatures were excluded. A narrower temperature range was used for NiO than for the other two metal oxides to avoid including the large mass loss at 550 °C. Table 3 summarizes the results for quantification before and after correction based on the mass loss for an unfunctionalized sample of the same size. Correction for the unfunctionalized sample decreases the estimated PVP content by ≤ 12% for the various samples.

Thermograms run in an air atmosphere showed predominant mass loss at ~ 400 °C for both Ce-PVP and Fe-PVP; the NiO samples both showed broader peaks between 400 and 450 °C, which probably reflects the presence of PVP and the contaminants observed for these samples. Representative thermograms are shown for Ce-PVP and Ni-PVP2 in Fig. S9. PVP content was estimated from the total mass loss between ~ 250 and 600 °C for each sample, before and after correction for an unfunctionalized sample run under air. As shown in Table 3 the results under argon and air vary with some values being higher in air and others in argon. It is possible that TGA measurements in air are more likely to give complete combustion of the adsorbed polymer and, therefore, more accurate quantification, than is obtained in an argon atmosphere. However, under air, the combustion of PVP may also be convoluted with loss of contaminants. For example, the broader mass loss peak for Ni-PVP2 in air (Fig. S9) compared to Ce-PVP could indicate contributions from surface contaminants observed for the unmodified sample. Nevertheless, comparison of the data for Ni-PVP2 with the known content (based on the amount of PVP used for surface coating) indicates that the corrected data measured under air is very close to the expected value. The argon-air comparison and the uncertainty related to correction for mass loss of components other than PVP highlight the potential issues associated with TGA quantification, even for samples with a relatively high loading of the surface coating.

### TGA of stearic acid-coated NPs

Representative TGA results for metal oxide NPs coated with stearic acid are shown in Figs. 7a and b and S10. There is a mass loss in the intermediate temperature range with 2 distinct peaks for Ce-SA1 and Ni-SA samples; the positions of the two peaks are slightly different for Ce-SA1 (310 °C and 440 °C) compared to Ni-SA (345 °C and 425 °C). The observation of two peaks with variable maxima and intensity is similar to previous observations for ZnO NPs coated with stearic acid. There are additional mass losses at ~ 200 °C for Ce-SA1 NPs and at higher temperatures for both CeO_2_ and NiO. The distinct peak at 200 °C varies in intensity for the three replicate runs for Ce-SA1 but is not observed for the other unfunctionalized or surface-modified CeO_2_ samples, including Ce-SA2, which is also modified with stearic acid. A similar mass loss was observed in thermograms in a nitrogen atmosphere for one of several surface-functionalized CeO_2_ nanoparticles in an earlier study, although an explanation was not provided [40].

FT-IR spectra for Ni-SA measured at 330 ℃ (Fig. 7c) showed signals consistent with a carbonyl at 1774 cm^−1^ and alkyl groups from 2800 to 3000 cm^−1^, providing corroborating evidence that this region can be assigned to loss of stearic acid. Note that the higher stearic acid content for this sample (based on the NMR quantification) allows detection of both carbonyl and alkyl signals consistent with stearic acid, whereas only the alkyl signals were detected previously for stearic acid-coated ZnO NPs. FT-IR spectra of evolved gases at 420 °C (Fig. 7d) also had a strong alkyl signal, but the carbonyl signal at 1774 cm^−1^ was no longer visible, although signals due to water in this region may interfere with a carbonyl signal.

The two Fe_2_O_3_ samples showed a different thermogram with a single peak at 380 °C and some mass loss above 650 °C (Fig. S10). The FT-IR spectrum at 380 °C showed predominantly CO_2_ loss. Although the mass loss occurs in a similar region to the other metal oxides, there is little evidence for a strong alkyl signal consistent with stearic acid. Based on the NMR results, Ni-SA has a higher stearic acid content than the other samples, which may explain why the FT-IR spectrum shows a strong alkyl group signal. It is possible that the results for stearic acid reflect two different modes of interaction with the metal oxide surface: carboxylate formation and adsorption of carboxylic acid. The FT-IR peak at 1774 cm^−1^ is consistent with a carbonyl group, indicating that the lower temperature peak may be due to stearic acid; the other peak is tentatively assigned to a carboxylate, which is expected to have C-O signals at lower wavenumber (e.g., 1544 to 1586 cm^−1^ for the asymmetric stretch for a number of metal stearates [41]).

XPS spectra of stearic acid-modified samples were examined to attempt to gain further insight on the TGA results. Examining the O1s peak shows that while Ni-SA exhibits evidence of extensive hydroxylation, as shown above for Ni-uf1 (unfunctionalized NiO NPs of the same size from the same supplier), the other two stearic acid-coated metal oxides exhibit normal levels of hydroxyl groups (~ 30% of the total O1s signal) with no adsorbed water (Fig. S11). A similar pattern is observed in the C1s region where Ni-SA NPs exhibit an increased fraction of C-O species compared with the Ce and Fe oxides. Since Ni and Ce both show two peaks in the TGA but different intensities in the C1s region, the XPS results do not provide insight on the identity of the two TGA peaks. We note that adventitious carbon contamination typically present for all nanomaterials handled in air prior to XPS measurements makes it difficult to draw quantitative conclusions regarding surface functionalization using the C1s region.

Thermograms recorded under air showed the main mass loss over a single narrow temperature range with peaks at 210 °C, 300 °C, and 270 °C for stearic acid-coated CeO_2_, NiO, and Fe_2_O_3_, respectively. In each case, there was a shoulder on the high temperature side of the main mass loss peak with different intensities for the various samples. Table 3 summarizes data for the quantification of stearic acid. The mass loss in the 350–430 °C region under argon with and without correction for an unfunctionalized sample of the same size is listed; both peaks are included for NiO and CeO_2_ samples. Under air, the mass loss for the main peak and the shoulder at higher temperature were used for quantification, also with and without correction.

### Method comparisons

A comparison of the qNMR and TGA data for the three metal oxide NPs modified with APTES, PVP, and stearic acid is shown in Fig. 8. In comparing the data, it should be first noted that our previous studies have shown that qNMR has high sensitivity, with an estimated limit of quantification of 10 µmol/g for the conditions used in the present study [7]. Extensive studies of the removal of aminopropyl siloxane groups (from APTES-modified NPs) have demonstrated good repeatability (typical relative standard deviations are ≤ 3%) in a single lab over an extended period of time, as well as good reproducibility (relative standard deviations ≤ 6%) in a bilateral comparison [7, 42]. Therefore, we conclude that the removal of siloxanes by hydrolysis followed by NMR quantification provides an accurate assessment of the functional group content. In this study, we have tested that functional groups can be efficiently removed for qNMR analysis by preparing samples with a known PVP and stearic acid content. Thus, we conclude that the qNMR data provide a repeatable and accurate assessment of the functional group content that can be used to assess the performance of TGA.

The comparison of results for APTES-modified NPs (Fig. 8a) provides both corrected (for mass loss in the region of the functional group for the corresponding unfunctionalized sample) and uncorrected TGA data. The qNMR and corrected TGA data (1055 and 1026 µmol/g) agree well for CeO_2_, which has the highest loading of functional group (expressed as a fraction of the total mass of the sample); a *t*-test indicates that the TGA and qNMR values are not significantly different. The TGA estimates for Ni-APTES and Fe-APTES are 1.3 and 2.4 times larger than the qNMR estimate, showing that agreement between the two methods decreases with lower surface functional group content. The corrected TGA estimate is closer to the qNMR data than the uncorrected estimate for each of the APTES-modified samples, indicating that the corrected data is more reliable, despite the fact that the manufacturer may not have prepared the surface-modified NPs from the same unfunctionalized sample that we used for correction. It should also be noted that the TGA data have not accounted for the ethanol observed in the qNMR spectra for these samples. If a fraction of the mass loss assigned to functional group is due to ethanol, this will yield a higher TGA estimate that may partly account for the poor agreement between TGA and qNMR data for Fe_2_O_3_ and NiO.

Previous studies for APTES-modified silica and ZnO NPs have reported TGA estimates (after correction for mass loss in an unfunctionalized sample) that were from two to five times higher than qNMR estimates for large NPs (80 and 100 nm; functional group content of 100–200 µmol/g) [16, 17]. The agreement between methods was worse for small NPs (20–30 nm) with low functional group content (< 50 µmol/g) but reasonably good (differences of 20% between the two methods) for small NPs with high surface loading (> 400 µmol). The correction is similar to or larger than the mass loss assigned to the functional group for most of these samples, contributing to the inaccuracy of the measurements. Based on the data presented here and previous work, one can conclude that TGA quantification for samples with a total content of 200–300 µmol/g should be sufficiently accurate, although the reliability of the result will depend on the availability of appropriate samples for correction. It is important to consider that the total functional group loading will be much larger for small NPs with a large surface area than for large NPs; therefore, one can anticipate more reliable results for small particles if the surface coverage is similar. However, the surface coverage can also vary significantly between batches of the same size NP, from the same supplier [7].

The comparison of qNMR and corrected TGA data measured under both argon and air for PVP-modified metal oxide NPs is shown in Fig. 8b. The TGA results measured under argon are within 20% of the qNMR value for the three samples with higher PVP content, but the agreement is lower for Fe_2_O_3_. However, a *t*-test indicates that only the mean values for Ce-PVP are different for the two methods. The TGA-air data is in better agreement with the qNMR data than the TGA argon data, although this is based on a single replicate for the measurements in air. The TGA-air-corrected value for Ni-PVP2 is in good agreement with the known amount of PVP used to produce the sample which is surprising since the TGA results indicate that the contaminant observed for the unfunctionalized NiO samples probably overlaps with the mass loss due to PVP. Although there does not appear to be any literature data for comparison for PVP-modified metal oxides, several PEG-modified silica NPs also showed good agreement between qNMR and TGA [16].

The comparison for stearic acid-modified samples shown in Fig. 8c indicates that the TGA data for air and argon are similar (with the possible exception of Ce-05); a *t*-test for Ce-SA1, Ni-SA, and Fe-SA1 indicates that the qNMR and TGA argon values are not significantly different. For three samples, the argon data are in closer agreement to the qNMR estimates, but the data do not provide a clear trend as to which is more reliable, even for the two samples prepared with a known amount of stearic acid. Overall, the agreement between TGA and qNMR is acceptable for stearic acid-modified NPs with loadings of ≥ 150 µmol/g; note that the higher molecular weight of stearic acid (compared to either APTES or the PVP monomer) accounts for the lower loading limit in micromole per gram.

The qNMR and TGA methods can be compared to literature studies using other techniques. A large number of studies from the Resch-Genger group have used optical probes to quantify surface accessible functional groups by absorption or emission spectroscopy and have frequently compared the results to the total functional group content measured by conductometric titration, NMR, or ICP-MS (for sulfur-containing probes) [9, 15, 26]. This work has focused primarily on polymer and silica NPs synthesized in their laboratory. A number of studies have used surface chemistry techniques such as XPS, EDS, and ToF–SIMS to study NP surfaces [4, 43]. These methods provide useful and complementary information to bulk methods such as TGA and qNMR of extracted functional groups’ methods as used here. However, there are challenges with ensuring that the sample preparation and the limited depth penetration and lateral resolution provide information that is representative of the entire sample; furthermore, the methods are less routinely available. Relatively few examples have probed surface chemistry for commercial nanomaterials. One notable exception is a recent study of a range of commercial nanomaterials (silica, silver, metal oxides, clays, carbon nanomaterials), some of which contained organic coatings [2]. Here, TGA was used to identify water and organic content and a combination of GC–MS, LC–MS, and MALDI-ToF–SIMS was used to provide identification and some quantification of organic coatings. Approximately 40% of the materials for which an organic coating was identified by TGA did not indicate the presence of extractable organics by MS. Both this study and our qNMR work require reliable methods for extraction of organic coating or release of covalently attached functional groups. The methods that have been developed and optimized here should be applicable to a range of metal oxides, an important consideration. Both studies illustrate the issues associated with TGA, despite the fact that this method has been relatively widely used, at least for qualitative measurements. Although the comparison of TGA in different atmospheres and coupling to FT-IR do overcome some limitations, this method is more limited that NMR or MS for structural identification, which is particularly important when the identity of the organic coating or functional group is not known or when multiple components are present.

## Conclusions

Both qNMR and TGA have been employed to quantify functional groups/coatings on the surface of three metal oxides, NiO, CeO_2_, and Fe_2_O_3_. The qNMR determinations rely on quantitative removal of the functional group or coating from the NP surface. An optimized hydrolysis method similar to that used previously for ZnO is suitable for removing aminopropylsilane from the NPs, demonstrating that this is a general approach for a range of metal oxides. Removal of PVP and stearic acid from the metal oxide NPs was accomplished using a ligand exchange method with sodium hexametaphosphate and PFDA, respectively. Although previous work had dissolved silica and zinc oxide NPs to remove functional groups for qNMR studies, the present approach avoids the complications associated with optimization of the metal oxide dissolution process. This is advantageous since the ease of dissolution for different metal oxides varies significantly [31]. The efficiency of functional group removal was verified by preparation of samples with known content of either stearic acid or PVP; this allows confirmation of quantitative removal of surface groups with a minimum number of trials of different conditions. The qNMR studies for NiO are complicated by the observation of partial dissolution releasing Ni ions that interfere with the qNMR studies in acidic solution, particularly in the presence of PFPA.

The qNMR data was used to evaluate the reliability of TGA experiments for the same samples in argon and air atmospheres, with FT-IR analysis of evolved gases under argon for selected samples. FT-IR provides confirmation of the temperature at which the functional groups are removed, which is particularly useful in cases where there are multiple components above 200 ℃. Comparison of results to unfunctionalized samples provides information on the mass loss due to contaminants or surface hydroxyls and can be supplemented with XPS measurements of surface composition. Although the presence of carbon contaminants precludes the use of XPS for assessing surface group content from C1s signals, it is interesting to note that XPS nitrogen content for APTES-modified samples agrees very well with the qNMR data. The results clearly show that correction of the TGA data for loss of components other than the functional group is important for most samples, although a correction is challenging to implement for commercial samples for which the sample history is unknown. Finally, it is not possible to generalize as to whether the estimates obtained for thermograms run under air or argon are more reliable, since the accuracy depends on the presence of contaminants and the availability of an appropriate unfunctionalized sample for comparison.

The data demonstrate that TGA gives reliable estimates of functional group content for samples with high surface loading, as discussed in the above section. The typical PVP and stearic acid contents in commercial samples are within the range where the TGA estimates agree well with the qNMR data. Therefore, TGA is a useful method for NPs modified with PVP (and presumably other polymers) or long-chain fatty acids. TGA may be more routinely available than qNMR, and in many cases, the better accuracy achievable with qNMR may not be essential. However, quantification of functional group loading is considerably more problematic for APTES-modified silicas or metal oxides where high surface loadings are needed to overcome the limitation of a low molecular weight functional group; practically speaking, this means that only small NPs (< 30 nm with close to monolayer coverages) are likely to be amenable to TGA. The present data and our previous work on silica and ZnO indicate that this is a general conclusion, although it should be noted that the problem is certainly worse for silicas, for which there is a large correction due to loss of surface hydroxyls in the same temperature range as the functional group [7].

In summary, this work developed general methods for removal of functional groups and coatings for three important types of surface-modified metal oxides. The availability of general approaches that can be applied to commercial materials is an important step. The study reinforces earlier conclusions on the use of TGA, and also indicates that its applicability can be expanded to NPs modified with either organic polymers or fatty acids. This begins to establish the range of surface coating content that can be assessed with acceptable accuracy by TGA. In this context, it is important to note that commercial suppliers generally do not provide any information on the content of functional groups or coatings. Furthermore, there is typically a lack of control of surface coverage, resulting in variable coverages even for different batches of the same size NP from the same supplier. These considerations highlight the importance of having reliable and easily implementable methods to estimate the surface content for both laboratory-synthesized and commercial nanomaterials. This will ultimately lead to improved quality control on nanomaterial properties, a development that will facilitate applications development as well as grouping and read across strategies for risk assessment.

## Supplementary Information

Below is the link to the electronic supplementary material.

Supplementary file1 (1.12 MB)
