# Supplementary material for: Surface chemistry of metal oxide nanoparticles: NMR and TGA quantification
Source: Anal Bioanal Chem. 2022 Mar 2;414(15):4409–25. doi: 10.1007/s00216-022-03906-x (PMC9142474; doi:10.1007/s00216-022-03906-x)
Supplement: Supplementary file 1 — Supplementary file1 (1.12 MB) [file 216_2022_3906_MOESM1_ESM.pdf]

# Supplementary Information

## Surface Chemistry of Metal Oxide Nanoparticles: NMR and TGA Quantification

Filip Kunc, Mary Gallerneault, Oltion Kodra, Andreas Brinkmann, Gregory P. Lopinski, Linda J. Johnston

National Research Council Canada, Ottawa, ON  K1A 0R6

Linda J. Johnston,  ORCID 0000-0002-9136-4920
linda.johnston@nrc-cnrc.gc.ca

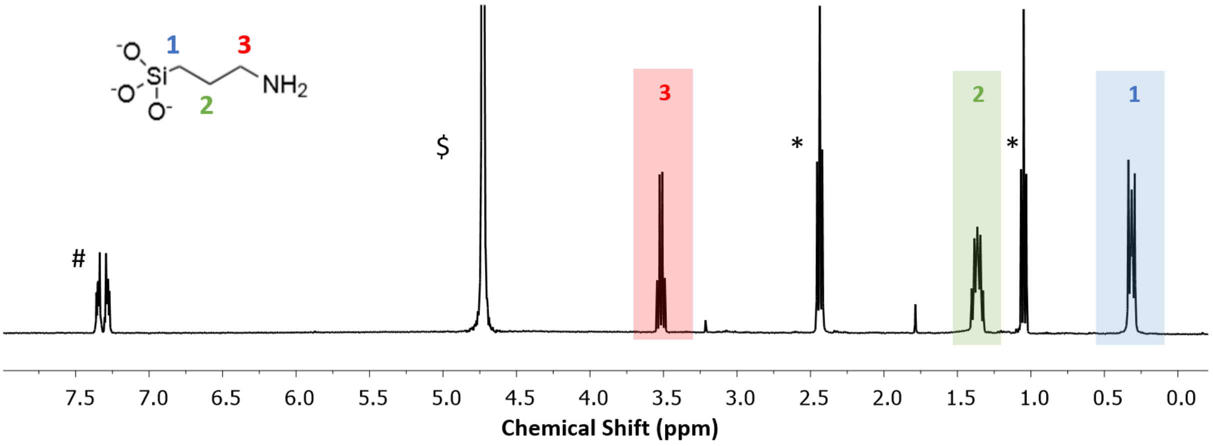


**Fig. S1**  The full $^{1}$H NMR spectrum of the sample (Ni-APTES) shown in Fig. 1 with quantification by comparison to the internal standard (#); note the signals for residual ethanol/alkoxy group (*), and the H-O signal ($). Signals for amine protons are not observed due to rapid exchange with $D_2O$.

Comparison of D2O wash and sodium hexametaphosphate (SHMP) ligand exchange for Ni-PVP1

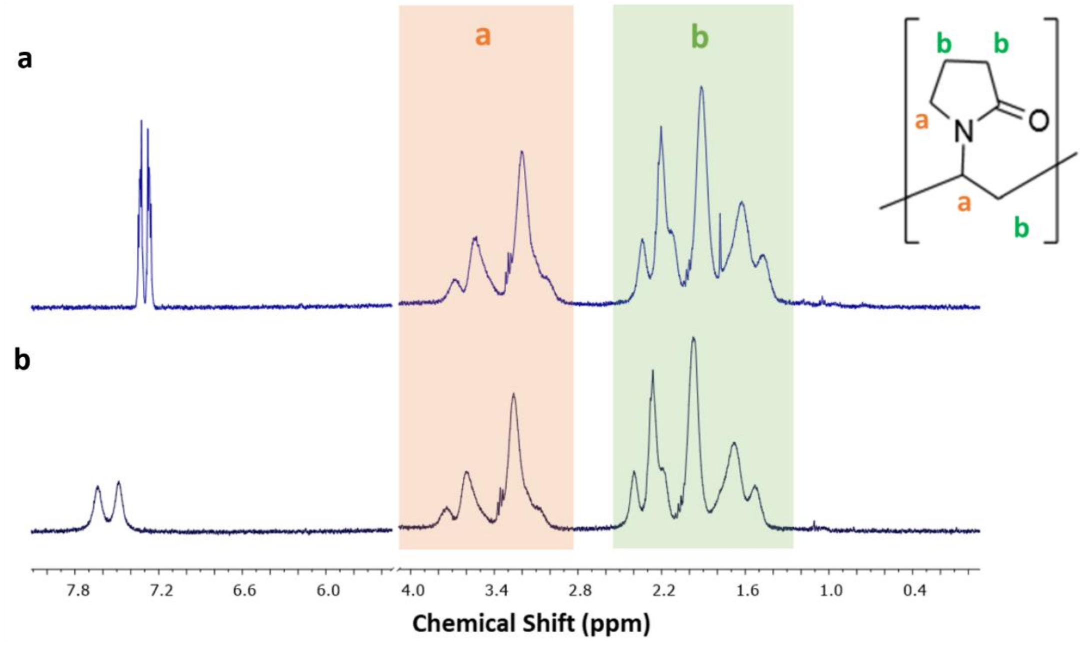


**Fig. S2** The $^{1}$H NMR spectra obtained using SHMP (a) and a $D_2O$ wash (b) to remove PVP from Ni-PVP1. Note the broadening of the internal standard and slight shift in the PVP signals in (b).

**Table S1** Quantification by qNMR of stearic acid removed from metal oxide nanoparticles (NPs) by solvent extraction in DMSO at 45 °C.

| Sample | Stearic acid, µmol/g | | |
|---|---|---|---|
| | 1st wash | 2nd wash | 3rd wash |
| Ce-SA1 | 5.9 | 4.9 | 7.3 |
| Ce-SA2[a] | 4.0 | | |
| Ni-SA | 115 | 44 | 46 |
| Fe-SA1 | 90 | 26 | 9 |
| Fe-SA2 | 53 | | |

[a] Functionalized with 300 µmol/g of stearic acid.

Summary of control experiments for NMR of NiO ligand exchange samples

The conditions at 45°C for 24 hours appear to cause 3,3,4,4,5,5,6,6,7,7,8,8,8-tridecafluorooctylphosphonic acid (PFPA) to dissolve the NiO surface and release ions into the analyzed solution. The observed broadening (Fig. S3) may be due to the interaction of PFPA with released ions. MeOD alone apparently does not etch/dissolve NiO, because no broadening is observed when PFPA is added to the supernatant consisting of MeOD that was exposed to NiO NPs alone. When stearic acid is added to the PFPA etched mixture, no shift is observed indicating that stearic acid is not involved in the interaction, although the alpha hydrogen signal interferes slightly with the broadened peaks of PFPA which may affect the accuracy during integration. Note that when maleic acid is added after the reaction (see Fig. S4) the signal is broadened similarly to signals for PFPA, suggesting that maleic acid is involved in the interaction with nickel ions.

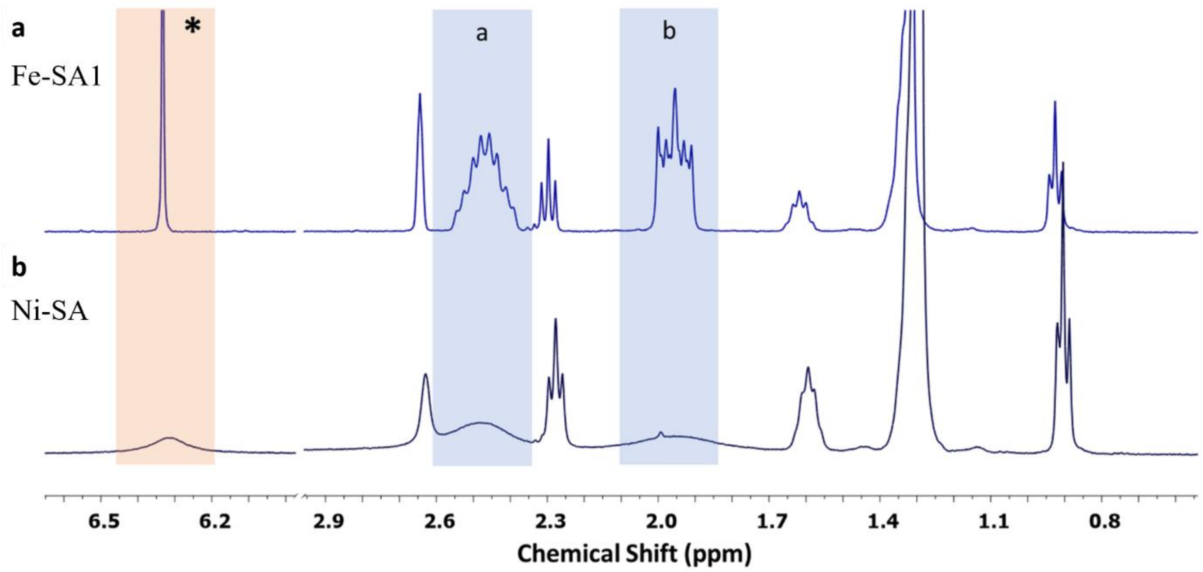


**Fig. S3** Representative NMR spectra of stearic acid coated metal oxides after ligand exchange with PFPA: (a) Fe-SA1, (b) Ni-SA. Note the broadening of the maleic acid signal (*), as well as the PFPA signals (a, b); interestingly, the stearic acid signals are not affected.

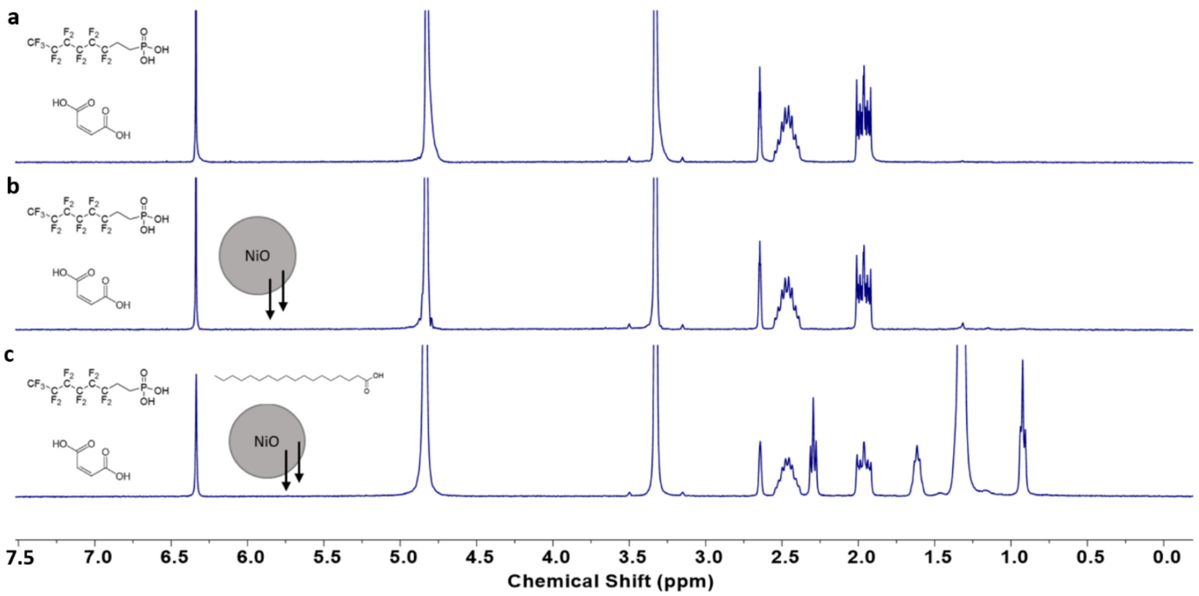


**Fig. S4** (a) Maleic acid mixed with PFPA at room temperature prior to NMR – no interaction; (b) Maleic acid combined with PFPA previously exposed to 2 mg of Ni-uf1 NPs. The intent was to observe if contamination by uncentrifuged nanoparticles can be the root cause of the effect. It is impossible to shim the magnet in the presence of the concentrated NP dispersion, and therefore, NPs were centrifuged for 10 s (as opposed to the 5 min interval typically used to remove NPs) and this spectrum of the supernatant was recorded – no interaction between components; (c) Maleic acid combined with PFPA, and stearic acid previously exposed to 2 mg of NI-uf1 NPs – no interaction between components.

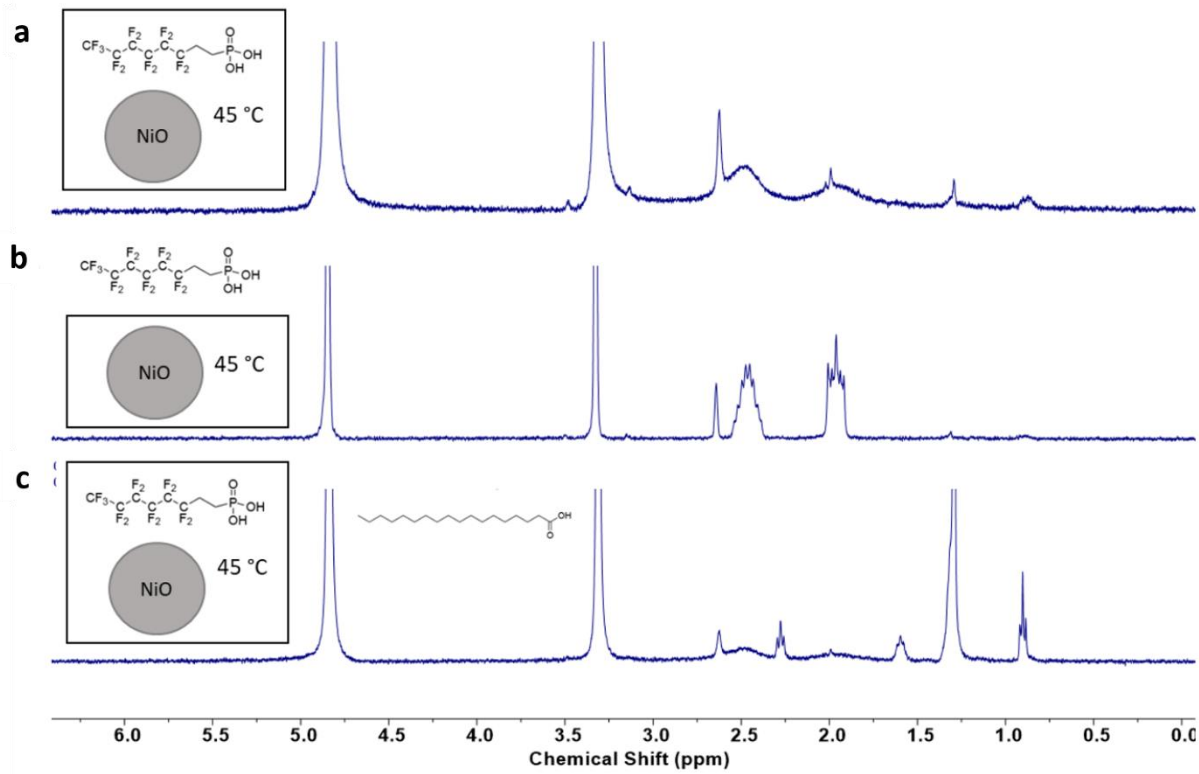


**Fig. S5** (a) Ni-uf1 shaken at 45 °C for 24 h in MeOD with PFPA – interaction occurs (b) Ni-uf1 shaken at 45 °C for 24 h in MeOD only and PFPA added right before the measurement – no interaction, (c) Ni-uf1 shaken at 45 °C for 24 h in MeOD with PFPA and stearic acid added prior to NMR – stearic acid signals are unaffected.

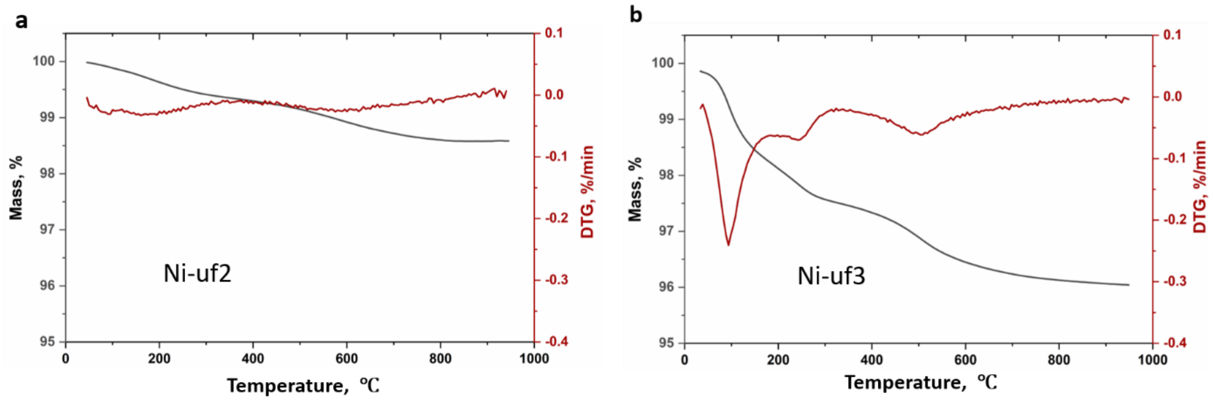


**Fig. S6** TGA results for Ni-uf2 (a) and Ni-uf3 (b) measured in an argon atmosphere.

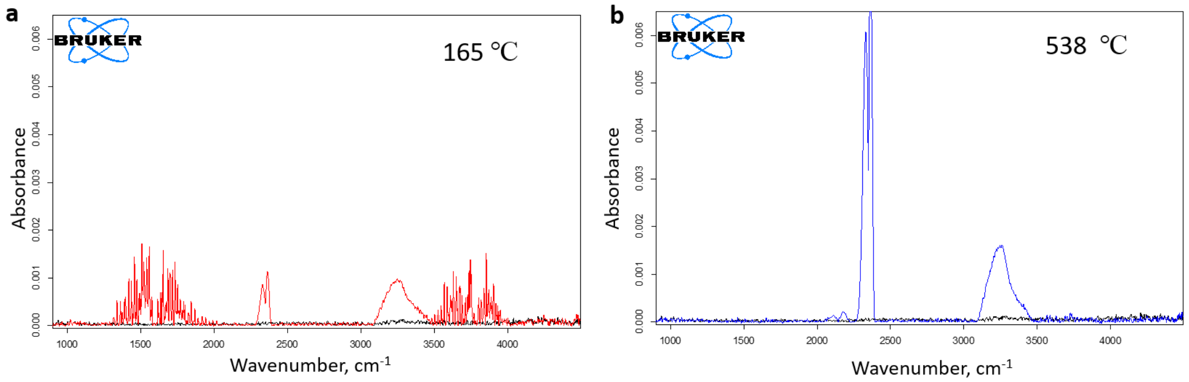


**Fig. S7** FT-IR spectra for Ni-uf1 (TGA in Figure 4) at 165 °C (a, predominantly water and $CO_2$) and 538 °C (b, predominantly $CO_2$). Note that the peak between 3000 and 3500 $cm^{-1}$ in both spectra is due to water condensation in the detector.

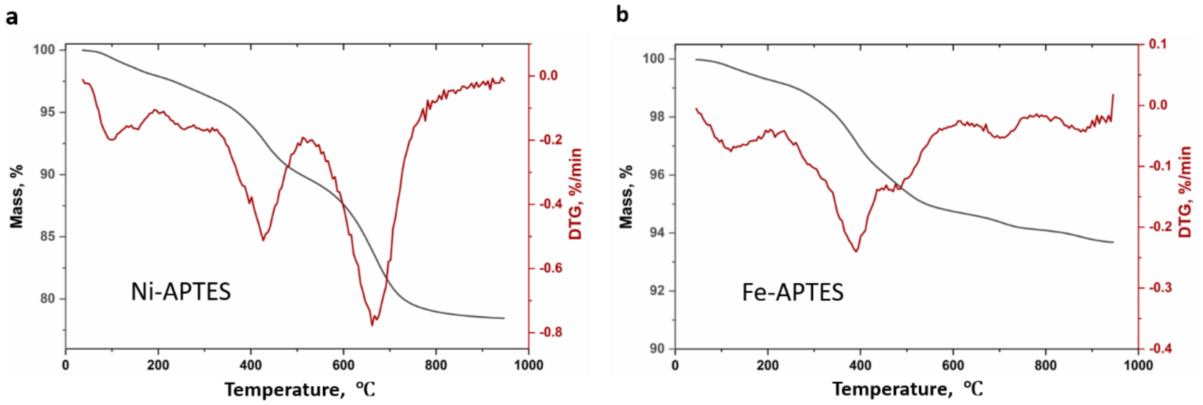


**Fig. S8** TGA results for Ni-APTES (a) and Fe-APTES (b) in an argon atmosphere.

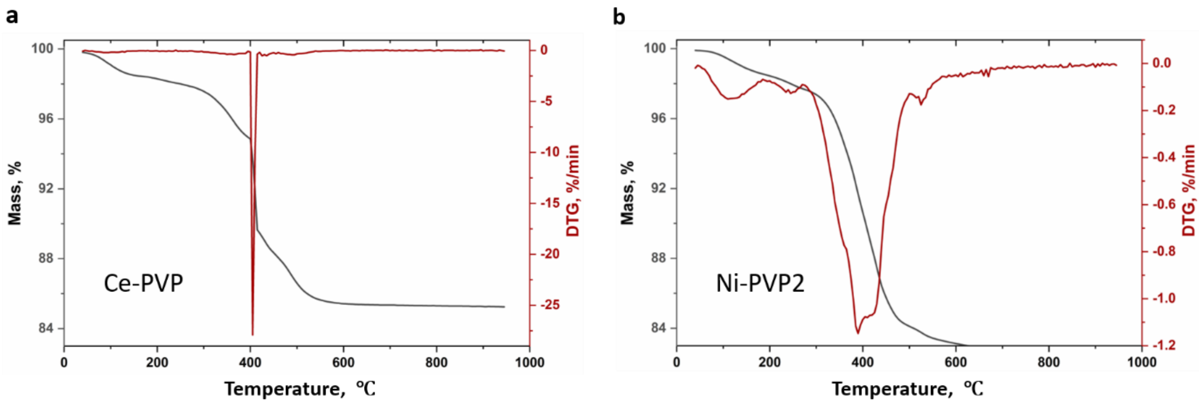


**Fig. S9** TGA results Ce-PVP (a) and Ni-PVP2 (b) in an air atmosphere.

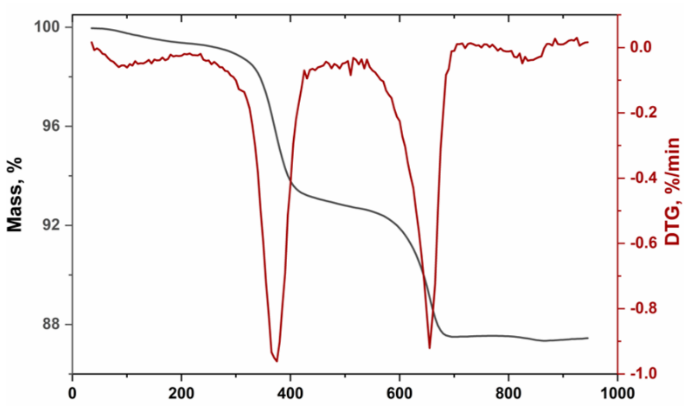


**Fig. S10** TGA results for Fe-SA1 in an argon atmosphere.

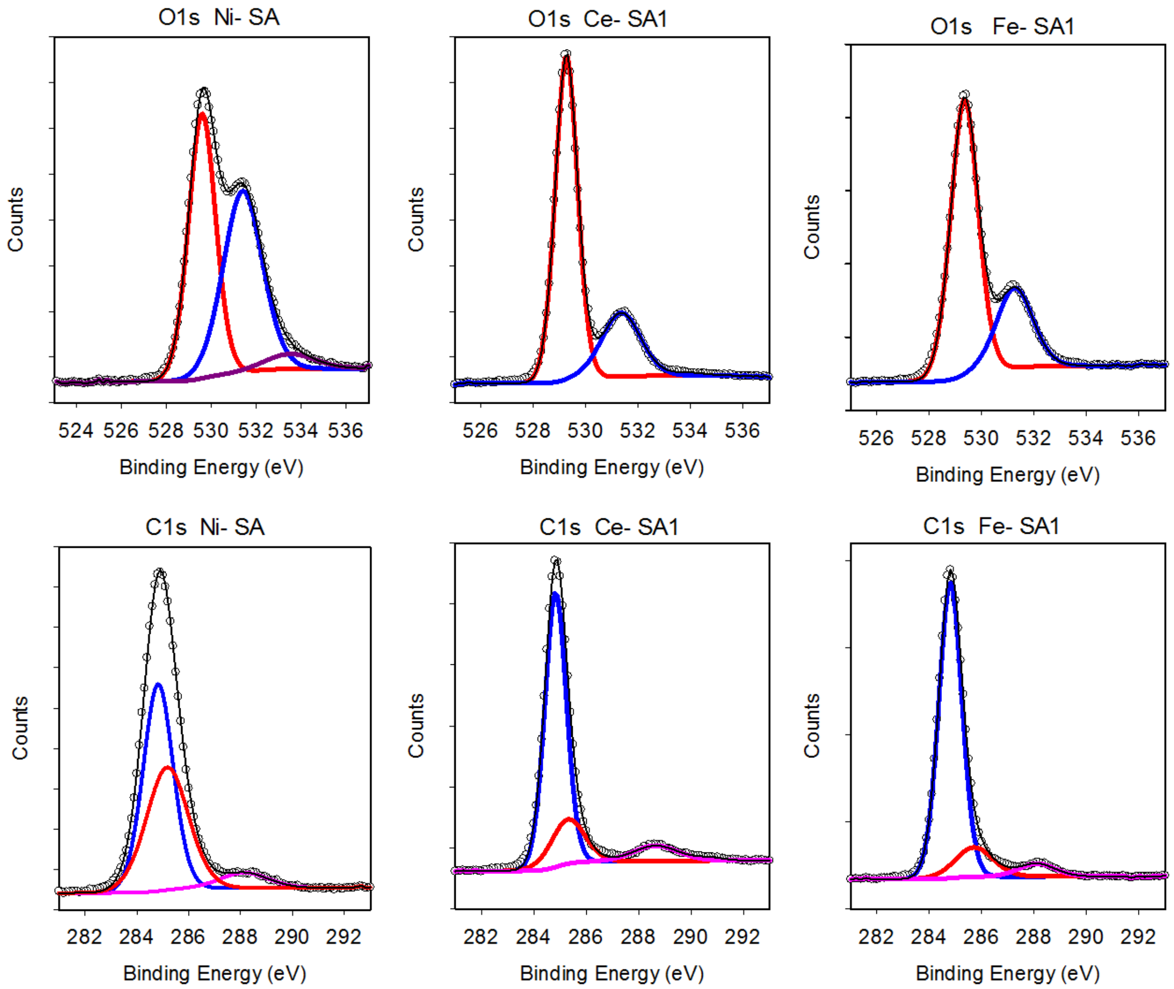


**Fig. S11** XPS data for O1s and C1s regions for Ni-SA, Ce-SA1 and Fe- SA1. The fits show the O1s peak decomposed into lattice oxygen (red), hydroxyl (blue) and adsorbed water (purple) with the water only visible for the Ni-SA sample. The C1s peak is decomposed into C-C (blue), C-O (red) and O-C=O (pink) components.
